# Geographically Associated Fungus-Bacterium Interactions Contribute to the Formation of Geography-Dependent Flavor during High-Complexity Spontaneous Fermentation

**DOI:** 10.1128/spectrum.01844-22

**Published:** 2022-09-22

**Authors:** Yuwei Tan, Hai Du, Hongxia Zhang, Chen Fang, Guangyuan Jin, Shuang Chen, Qun Wu, Yan Zhang, Menghui Zhang, Yan Xu

**Affiliations:** a Laboratory of Brewing Microbiology and Applied Enzymology, School of Biotechnology, Jiangnan University, Wuxi, Jiangsu, China; b Key Laboratory of Industrial Biotechnology of Ministry of Education, Jiangnan University, Wuxi, Jiangsu, China; c Bioprocess Engineering, Wageningen University and Research, Wageningen, The Netherlands; d Key Laboratory of Systems Biomedicine (Ministry of Education), Shanghai Center for Systems Biomedicine, Shanghai Jiao Tong University, Shanghai, China; e State Key Laboratory of Microbial Metabolism, Shanghai Jiao Tong University, Shanghai, China; f Joint International Research Laboratory of Metabolic and Developmental Sciences, School of Life Sciences and Biotechnology, Shanghai Jiao Tong University, Shanghai, China; Broad Institute

**Keywords:** Chinese liquor, flavor biotechnology, geography-dependent flavor, microbial community, microbial interaction, spontaneous fermentation

## Abstract

Fermented foods often have attractive flavor characteristics to meet various human demands. An ever-challenging target is the production of fermented foods with equal flavor profiles outside the product’s origin. However, the formation of geography-dependent flavor in high-complexity fermentations remains poorly understood. Here, taking Chinese liquor (baijiu) fermentation as an example, we collected 403 samples from 9 different locations in China across a latitude range of 27°N to 37°N. We revealed and validated the geography-dependent flavor formation patterns by using culture-independent (metabolomics, metagenomics, and metatranscriptomics) and culture-dependent tools. We found that the baijiu microbiomes along with their metabolites were flavor related and geography dependent. The geographical characteristics were determined mainly by 20 to 40 differentiated chemical markers in metabolites and the latitude-dependent fungal structure of the microbiome. About 48 to 156 core microbiota members out of 735 bacterial genera and 290 fungal genera contributed to the chemical markers. The contributions of both fungi and bacteria were greater than those from either bacteria or fungi alone. Representatively, we revealed that dynamic interdependent interactions between yeasts and *Lactobacillus* facilitated the metabolism of heterocyclic flavor chemicals such as 2-acetylpyrrole, 2,3,5-trimethylpyrazine, and 2-acetylfuran. Moreover, we found that the intraspecific genomic diversity and microbial structure were two biotic factors that contributed to dynamic microbiome assembly. Based on the assembly pattern, adjusting the composition and distribution of initial species was one option to regulate the formation of diverse flavor characteristics. Our study provided a rationale for developing a microbiome design to achieve a defined flavor goal.

**IMPORTANCE** People consume many spontaneously fermented foods and beverages with different flavors on a daily basis. One crucial and hotly discussed question is how to reproduce fermented food flavor without geographical limitations to meet diverse human demands. The constantly enriched knowledge of the microbial contribution to fermented flavor offers valuable insights into flavor biotechnological development. However, we still have a poor understanding of what factors limit the reproduction of fermented flavor outside the product’s origin in high-complexity spontaneous fermentations. Here, taking baijiu fermentation as an example, we revealed that geography-dependent flavor was contributed mainly by fungus-bacterium cooperative metabolism. The distinct initial microbial composition, distribution, and intraspecific genomic diversity limited reproducible microbial interactions and metabolism in different geographical areas. The abundant microbial resources and predicted fungus-bacterium interactions found in baijiu fermentation enable us to design a synthetic microbial community to reproduce desired flavor profiles in the future.

## INTRODUCTION

Fermented foods accompany human civilization and development not only because of the primary purpose for food preservation but also for the many attractive and distinguishing flavors (1, 2). Fermented foods from different geographical areas often have unique flavor characteristics compared to similar products from other areas. For example, regionally distinct wine flavor characteristics are widely known as terroir (3, 4). Such geographical flavor characteristics can be profiled by chemical compositions (5, 6). It is always of great interest to produce fermented foods with equal flavor profiles regardless of the geographical difference of the product’s origin (7–10). Therefore, revealing how distinct chemical compositions are formed will help reproduce diverse food flavors without geographical limitations to meet wider human demands (11).

So far, researchers have clarified the key causes of geography-dependent flavor formation in less complex food fermentations (wine, soy sauce, and cheeses, etc.). Geographical microbial variations, phage infection, facility-specific parameters, and local environments (temperature, precipitation, humidity, and water quality, etc.) are essential for the generation of flavor-related chemicals (1, 12–14). For example, the quality of wine from the different vineyards is conditioned by the grape microbiome, which is related to geographical microbial strains (3, 15). The wine grapes are then transformed into wine through microbial activity, with indisputable consequences for wine chemical compositions (4). In the case of soy sauce, Japanese soy sauce with the same quality can now be produced in the United States and Europe by applying pure cultures of Koji and the same processes as the ones used in Japan (1). As for cheese fermentation, microbial strains from the United States and Europe with different CRISPR spacers or metabolic genes can affect the pigmentation of the cheese and the production of aroma compounds (12, 16). However, in high-complexity spontaneous food fermentations, the mechanism of geography-dependent flavor formation is not yet completely understood, resulting in difficulties in standardization and modernization (17).

**FIG 1:**
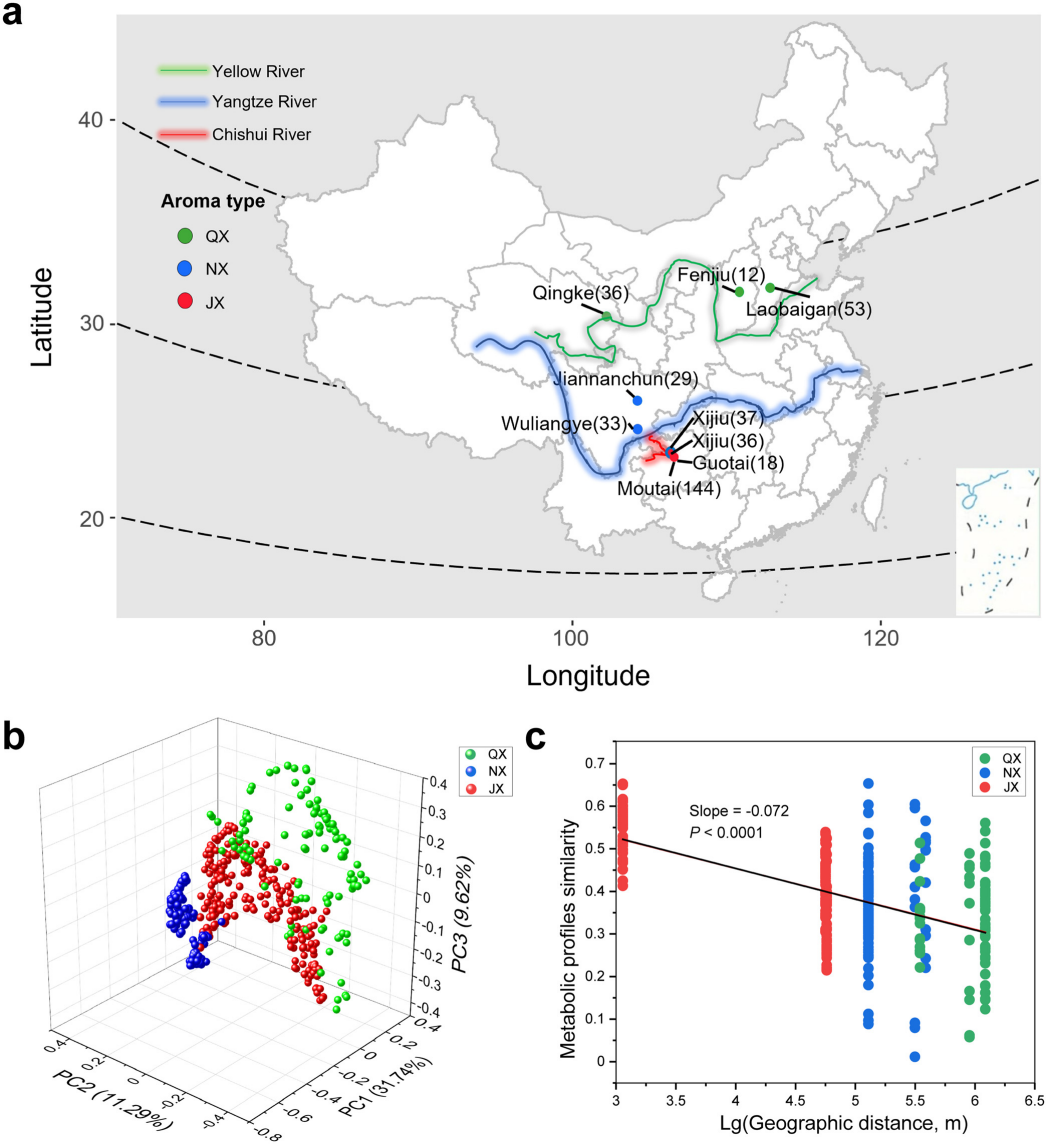
Geography-dependent flavor characteristics of baijiu fermentations. (a) Distribution of three typical distilleries (QX, Qingxiang [light aroma]; JX, Jiangxiang [sauce aroma]; NX, Nongxiang [strong aroma]) in China. Valleys are marked by lines, and sampling sites are marked by points. The color of the point indicates the aroma type produced by the distillery. The numbers in parentheses indicate the sample size. (b) Principal-component analysis plot showing the distinct volatile metabolite profiles among 403 samples of JX, NX, and QX. (c) The similarity of volatile chemical profiles among the 403 samples is significantly related to the corresponding geographical distance. The geographical distance was converted by log.

Among high-complexity food fermentations, Chinese liquor fermentation is a representative complex process with saccharification and spontaneous fermentation simultaneously (18), and more particularly, fermentation is under solid-state conditions (19). Researchers revealed that Chinese liquor can be produced with different cultivars of sorghum (raw materials) from China, America, or Australia (20, 21). In addition, Chinese liquor can also be produced with different production processes (22). As a result, several geographically distinct flavor types appeared under regionally preferred fermentation types despite the similar raw materials used (mainly Chinese sorghum) (23). Among them, three typical geography-dependent aroma types, named Qingxiang (QX) (light-aroma type), Nongxiang (NX) (strong-aroma type), and Jiangxiang (JX) (soy aroma type), have been documented (Fig. 1a). Microbial activity was an integral part of Chinese liquor production. However, the large pool of microbial geographical diversity, the many uncharacterized microbial metabolic interactions, and the limited approaches available to regulate flavor formation still make it a challenge to reproduce aroma types in different geographical areas (17, 22, 24–27).

To further understand the mechanism of geography-dependent flavor formation, we explored Chinese liquor fermentation to assess (i) the geographical characteristics of the chemical and microbial compositions, (ii) why microbial geographical diversity can contribute to distinct flavor formation; and (iii) how to regulate the microbial generation of flavor-related chemicals. Here, we collected 403 samples of baijiu fermentations from 9 different locations in China across a latitude range of 27°N to 37°N, belonging to 3 aroma types of Chinese liquor (JX, NX, and QX). We used gas chromatography-mass spectrometry (GC-MS) to measure the volatile metabolites of fermented samples. In addition, marker metabolites of flavor types at geographical scales were identified by partial least-squares regression discriminant analysis. We used high-throughput amplicon sequencing to profile the bacterial and fungal geographical diversity of fermented samples. Microbial geographical diversity was assessed at the latitude and longitude levels. We then revealed the distinct associations of microbial genera with marker metabolites by molecular ecological networks in three groups. Furthermore, we clarified the molecular mechanisms of partial associations in the JX group. Time series metatranscriptome sequencing was used to illustrate pathways and genes that contribute to the generation of marker metabolites during the fermentation process. What is more, we identified the trigger factors underlying the distinct microbiome assemblies by comparing the initial microbial structure and the initial genomic diversity. Metagenome sequencing and metagenome-assembled genomes (MAGs) were used to assess microbial genomic diversity. Based on trigger factors, we then validated two optional approaches for regulating flavor formation. Our findings provide novel ecological and molecular insights into the formation of geography-dependent flavor in high-complexity spontaneous food fermentations and advance our ability to develop a microbiome design to achieve a defined flavor goal.

## RESULTS

### Geographical characteristics of fermented chemical compositions.

In total, GC-MS detected 471 volatile compounds (not including ethanol) from 403 baijiu fermented samples that may contribute to baijiu flavor. Among 471 volatiles, 56 metabolites were shared by all three groups (QX, NX, and JX) in the samples. We found 69 unique volatile chemicals in QX, 206 in NX, and 62 in JX (see Fig. S1a in the supplemental material). The main chemicals included esters, alcohols, acids, aldehydes, and multiple aromatic compounds (Data Set S1). Based on the relative concentrations of metabolites at the end of the fermentation, esters were the same most abundant volatile metabolites in QX (89%), NX (92%), and JX (73%). Besides, NX and JX contained more acids than QX, while QX and JX contained more alcohols than NX (Fig. S1b). Based on the relative concentration of metabolites during the whole fermentation process, a principal-component analysis plot shows clear clusters of samples (Fig. 1b). We found that the difference among the three groups was greater than that within each group by analysis of similarity (ANOSIM) (*R *= 0.714; *P < *0.001).

The fermented chemical compositions were geography dependent (tested by one-way analysis of variance [ANOVA] [*P < *0.0001]). We found a significant linear correlation between metabolic profiles and geographical distance (Fig. 1c). The typical QX distilleries were located mainly at a latitude range of 36°N to 37°N next to the Yellow River. The typical NX distilleries were located primarily at a latitude range of 28°N to 30°N next to the Yangtze River (Fig. 1a). The typical JX distilleries were located mainly at a latitude range of 27°N to 28°N next to the Chishui River. Notably, the longitude range of JX (106.1°E to 106.4°E) was much smaller than those of NX (104.1°E to 106.2°E) and QX (101.9°E to 115.7°E).

We confirmed the significant geographical difference in volatile chemical compositions among the three groups by partial least-squares regression discriminant analysis (*Q*^2^ = 0.902, *R*^2^*Y* = 0.930, and *Q*^2^*Y* = 0.896). Table S2 shows the contribution and confidence of each chemical variable from the established partial least-squares model. We found that the different geographical characteristics of chemical compositions were contributed mainly by 81 flavor-related metabolites (variable importance in projection [VIP] value of >1). The 81 flavor-related metabolites contained 37 esters, 11 alcohols, 9 acids, 7 aldehydes, and 17 other chemicals. For these 81 chemicals, QX samples presented 54 flavor-related metabolites, including several long-carbon-chain alcohols, such as 3-methyl-1-butanol, 3-methylthiopropanol, 1-nonanol, and 3-octanol. JX samples presented 53 flavor-related metabolites, mainly short-carbon-chain acids, such as propanoic acid, butanoic acid, and 3-methylbutanoic acid. NX samples exhibited high relative concentrations of 62 flavor-related metabolites, primarily esters, such as ethyl caproate, ethyl butyrate, ethyl caprylate, ethyl valerate, ethyl 3-phenylpropionate, propyl caproate, and ethyl heptanoate (Fig. S2). Table 1 shows the geographical chemical markers of the three aroma types after filtering 81 metabolites based on the majority (50%) within each group.

**TABLE 1:** Main chemical markers of fermented grains among the three aroma types of baijiu

Chemical taxon	Chemical marker		
	QX	JX	NX
Esters	Ethyl laurate	Ethyl lactate	Ethyl caproate
	Ethyl myristate	Ethyl phenylacetate	Ethyl butyrate
	Ethyl oleate	Ethyl nonanoate	Ethyl caprylate
	Ethyl palmitate		Ethyl valerate
	Ethyl pentadecanoate		Ethyl 3-phenylpropionate
	Diethyl succinate		Propyl caproate
	Ethyl benzoate		Ethyl heptanoate
	Ethyl linoleate		Hexyl acetate
	Isoamyl lactate		
	Methyl 2-methyltetradecanoate		
	2-Propenyl phenylacetate		
	Ethyl heptadecanoate		
	Ethyl octadecanoate		
	Ethyl linolenate		
	Ethyl 3-methylbutanoate		

Alcohols	3-Methyl-1-butanol	1,2-Propanediol	
	Isobutanol	Furfuryl alcohol	
	3-Methylthiopropanol	Benzyl alcohol	
	1-Nonanol	2,3-Butanediol	
	3-Octanol	β-Ethylphenethyl alcohol	

Acids	Octanoic acid	Propanoic acid	Hexanoic acid
		3-Methylbutanoic acid	
		Benzoic acid	
		Butanoic acid	

Aldehydes	1-Nonanal	Benzeneacetaldehyde	
	Hexanal		
	(*E*)-2-Octenal		
	Acetaldehyde		
	3,5-Dimethylbenzaldehyde		

Others	Styrene	2,3,5-Trimethylpyrazine	*p*-Cresol
	2-Methoxy-3-(2-methylpropyl)pyrazine	Tetramethylpyrazine	
	Naphthalene	2-Pentyl furan	
	Damascenone	2-Acetyl-5-methylfuran	
	Butylated hydroxytoluene	Acetophenone	
	2,3-Dihydrobenzofuran	2-Acetylpyrrole	
	4-Ethylphenol	1-(Furan-2-yl)ethanone	
	2-Methoxy-4-vinylphenol		

### Geographical characteristics of the baijiu microbiome.

The microbial taxonomic profiles of 403 baijiu fermentation samples were analyzed to clarify the microbial geographical characteristics. We classified 811 bacterial amplicon sequence variants (ASVs) in QX, 1,851 bacterial ASVs in NX, and 3,032 bacterial ASVs in JX. The numbers of fungal ASVs were 648, 832, and 1,108 in QX, NX, and JX, respectively. In total, we annotated 735 bacteria and 290 fungi to the genus taxonomic level within the baijiu fermentation ecosystem (Table S3).

**FIG 2:**
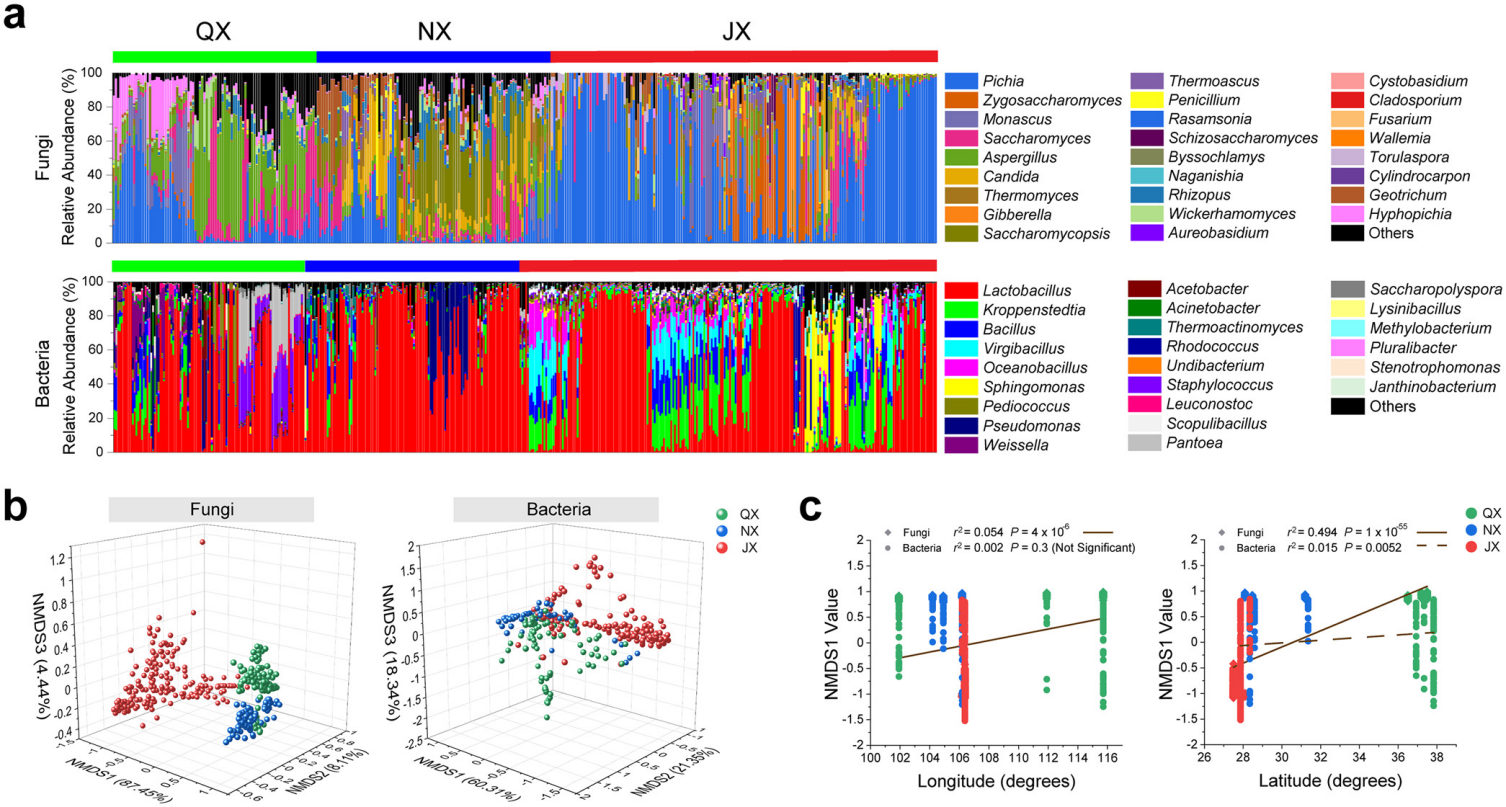
Microbial characteristics of QX, NX, and JX in 403 samples. (a) Genera that were detected at a relative abundance of ≥0.1% in this survey (see Data Set S2 in the supplemental material). (b) Nonmetric multidimensional scaling (NMDS) plot based on the structures of the bacterial (left) and fungal (right) communities. (c) Microbial NMDS1 is significantly linked to geographic longitude and latitude. The lines represent the regression line fitted by the first-order polynomial.

We found that the most abundant (relative abundance of >0.1%) microbial genera were shared among geography-dependent aroma groups, comprising over 90% of the total bacterial or fungal relative abundance (Fig. 2a). For QX samples, *Lactobacillus*, *Weissella*, Pseudomonas, *Bacillus*, and *Pediococcus* were the most abundant bacteria, whereas *Saccharomycopsis*, *Saccharomyces*, *Candida*, *Pichia*, and Aspergillus were the most abundant fungi. For NX samples, *Lactobacillus*, Pseudomonas, and *Bacillus* were the most abundant bacteria, whereas *Pichia*, *Kazachstania*, and Aspergillus were the most abundant fungi. For JX samples, *Lactobacillus*, *Kroppenstedtia*, *Bacillus*, and *Lentibacillus* were the most abundant bacteria, whereas *Issatchenkia*, *Pichia*, *Byssochlamys*, *Thermomyces*, and *Saccharomyces* were the most abundant fungi. Interestingly, the dominant genera were similar among the three groups but with different relative abundances, such as *Lactobacillus*, *Saccharomyces*, and some non-*Saccharomyces* yeasts (Data Set S2). Rare (relative abundance of <0.1%) microbial genera comprised over 90% of the unique bacterial or fungal genera. JX included the largest number of unique bacterial and fungal genera in fermented grains, followed by NX and QX (Data Set S2 and Fig. S3). Statistically, the three groups represented significantly different (*P < *0.001) microbial structures at the genus taxonomic level (Fig. 2b). For fungal structures, the JX group was separated from the QX and NX groups on nonmetric multidimensional scaling 1 (NMDS1). In addition, the NX and QX groups were separated on NMDS3. The two axes explained 87.45% and 4.44% of the total variance in fungal community differentiation in QX, NX, and JX. The separation by bacterial genera among the three groups was less distinct than that by fungi, indicating that the differences in fungal structures might be larger.

Both fungal and bacterial structures were significantly related to latitude rather than longitude (Fig. 2c). The fungal structure exhibited more significant linear correlations with latitude (*R*^2^ = 0.494; *P < *0.001) than with longitude (*R*^2^ = 0.054; *P < *0.001), indicating that fermentation fungal constituents were latitude dependent (Fig. 2c and Fig. S4). The bacterial structure showed a weak (*R*^2^ = 0.002; *P < *0.001) correlation with longitude. Besides, the correlation between the bacterial structure and longitude was insignificant, indicating that fermentation bacterial genera were not geographically dependent.

### Interactions between geographical chemical markers and the baijiu microbiome.

We found that both bacteria and fungi contributed to chemical markers. Table S4 shows that bacteria and fungi were significantly correlated with volatile chemicals (*P < *0.01 by a Mantel test). We modeled the associations between them individually in the three groups through two-way orthogonal partial least-squares (O2-PLS) modeling. The *Q*^2^ values of the models were 0.375 for the QX group, 0.219 for the NX group, and 0.262 for the JX group. In total, we identified 212 bacterial genera and 106 fungal genera as volatile chemical-associated microbial genera in QX, 79 bacterial genera and 69 fungal genera in NX, and 97 bacterial genera and 39 fungal genera in JX (Data Set S3). We then characterized the core microbial genera that contribute to geographically flavor-related chemical markers. After filtering microbial genera based on coverage in the samples (>20%), it was determined that 46 chemical markers were significantly associated with 69 core bacteria and 87 core fungi in QX, 44 chemical markers were significantly associated with 39 core bacteria and 34 core fungi in NX, and 35 chemical markers were significantly associated with 35 core bacteria and 13 core fungi in JX (Data Set S3).

**FIG 3:**
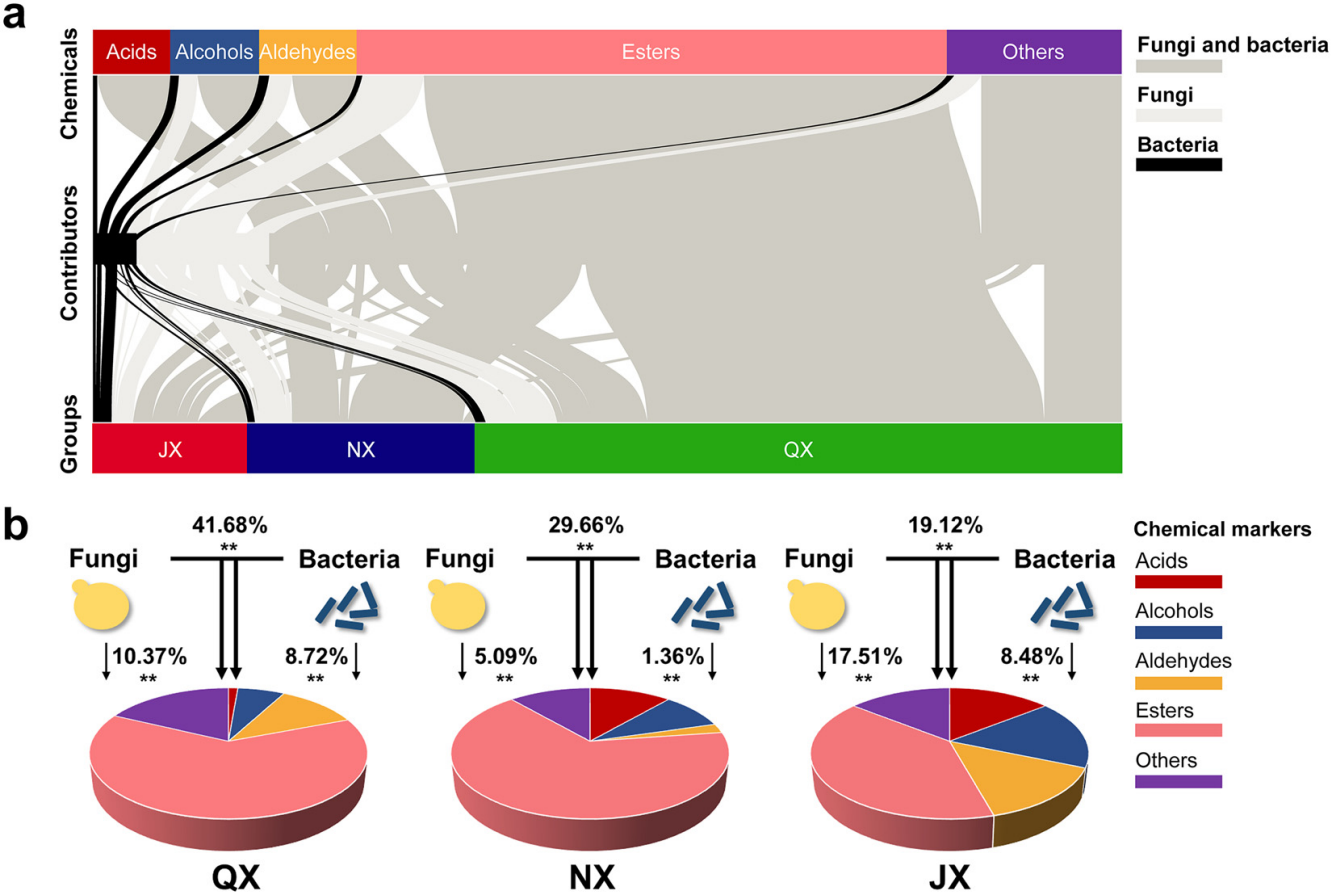
Contribution patterns of flavor-related chemical markers. (a) Interrelationship between chemical markers and microbial contributors at the chemical classification level and the group classification level. Shown is a visualization of the significant correlation network according to Pearson correlation analysis between normalized chemical concentrations and microbial relative abundances. (b) Variation partition analysis of the effects of fungi, bacteria, and fungus-bacterium interactions on the metabolic profiles of chemical markers. Percentages are variance explanation of the three factors.

Our results showed that 87.3% of the core fungal and bacterial genera participated in the cometabolism of chemical markers in the QX group, 80.5% participated in the NX group, and 74.1% participated in the JX group (Fig. 3a). Meanwhile, most acids, alcohols, aldehydes, esters, and other aromatic compounds were contributed by both fungi and bacteria. Figure 3b reveals that the contribution of fungus-bacterium cometabolism to geography-dependent flavor was greater than that from either bacteria or fungi solely. The variance explanation of cometabolism are 41.68% in the QX group, 29.66% in the NX group, and 19.12% in the JX group (Fig. 3b).

Figure S5 shows detailed significant associations between chemical markers and the core microbial community at the phylum and genus taxonomic levels. The Ascomycota, Basidiomycota, Zygomycota, and Mucoromycota were the fungal phyla that contributed to chemical markers. The *Firmicutes* and *Proteobacteria* were the same top two bacterial phyla that contributed to chemical markers in the three groups (Fig. S5a). Generally, the QX and NX groups showed more redundant associations between aldehydes and core microbial genera than did the JX group. The NX and JX groups showed more redundant associations between acids and core microbial genera than did the QX group (Fig. S5b).

**FIG 4:**
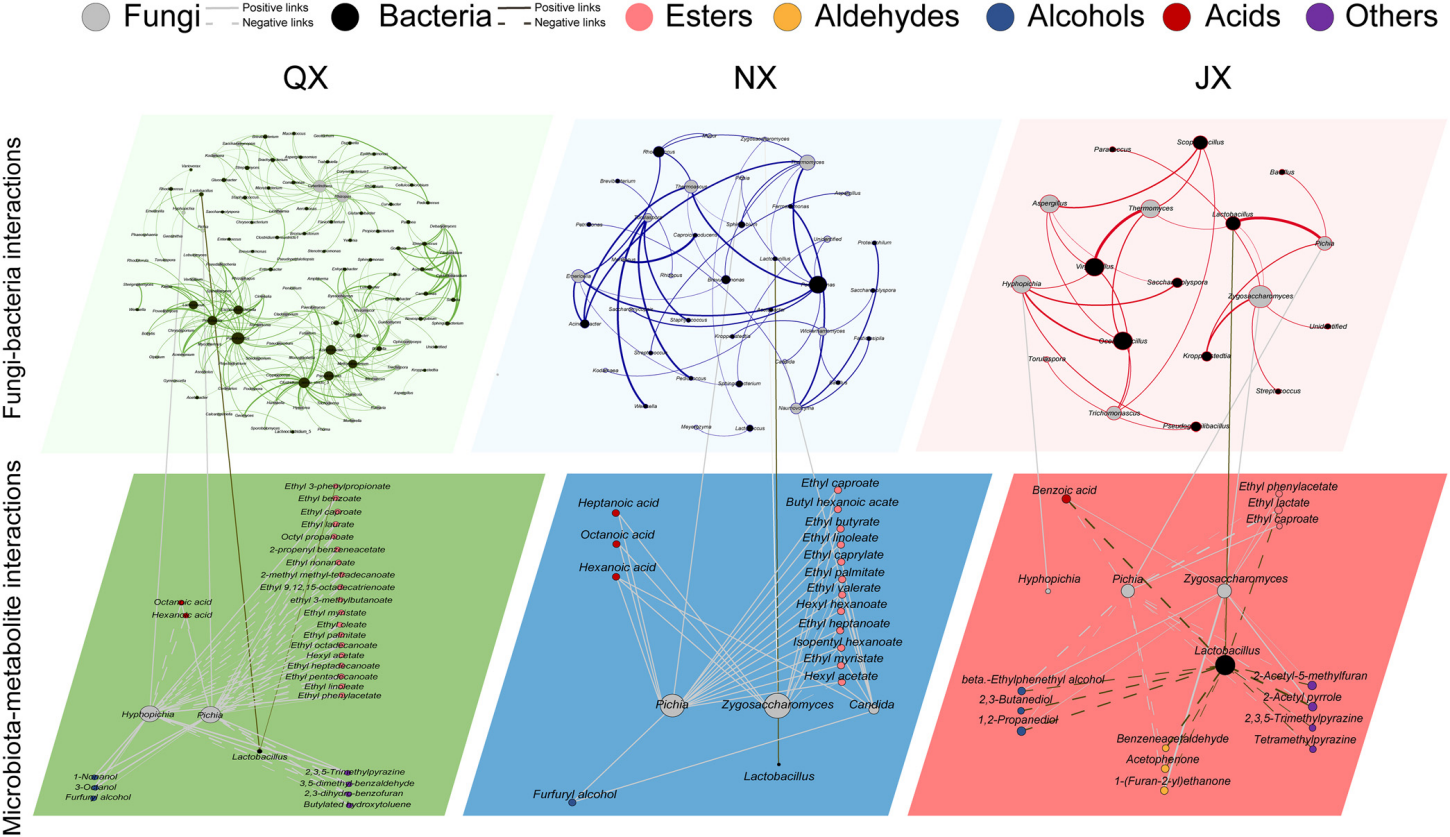
The identified core fungus-bacterium interactions and patterns of contribution to the volatile chemical markers during QX, NX, and JX fermentations. The core bacteria and fungi along with their corresponding relationships with the metabolite markers individually screened by O2-PLS modeling are shown (see Data Set S3 in the supplemental material). Only microbiotas with high coverage (>50%) are shown. The green, blue, and red lines represent microbial interactions within the QX, NX, and JX groups, respectively.

Notably, the same core fungal and bacterial genera showed different fungus-bacterium and microbiota-metabolite interactions among the three groups (Fig. 4). In the QX group, the interactions among *Hyphopichia*, *Pichia*, and *Lactobacillus* were related mainly to esters such as ethyl 3-phenylpropionate, ethyl palmitate, and ethyl octadecenoate. In the NX group, the interactions among *Pichia*, Zygosaccharomyces, *Candida*, and *Lactobacillus* were related primarily to esters and acids such as hexanoic acid, butyl hexanoate, and ethyl caproate. In the JX group, the interactions among *Pichia*, Zygosaccharomyces, and *Lactobacillus* were significantly related to diverse chemicals such as 2-acetylpyrrole, tetramethylpyrazine, β-ethylphenethyl alcohol, and benzoic acid (Fig. 4 and Fig. S5b).

### Fungus-bacterium dynamic cometabolism of geographical chemical markers.

**FIG 5:**
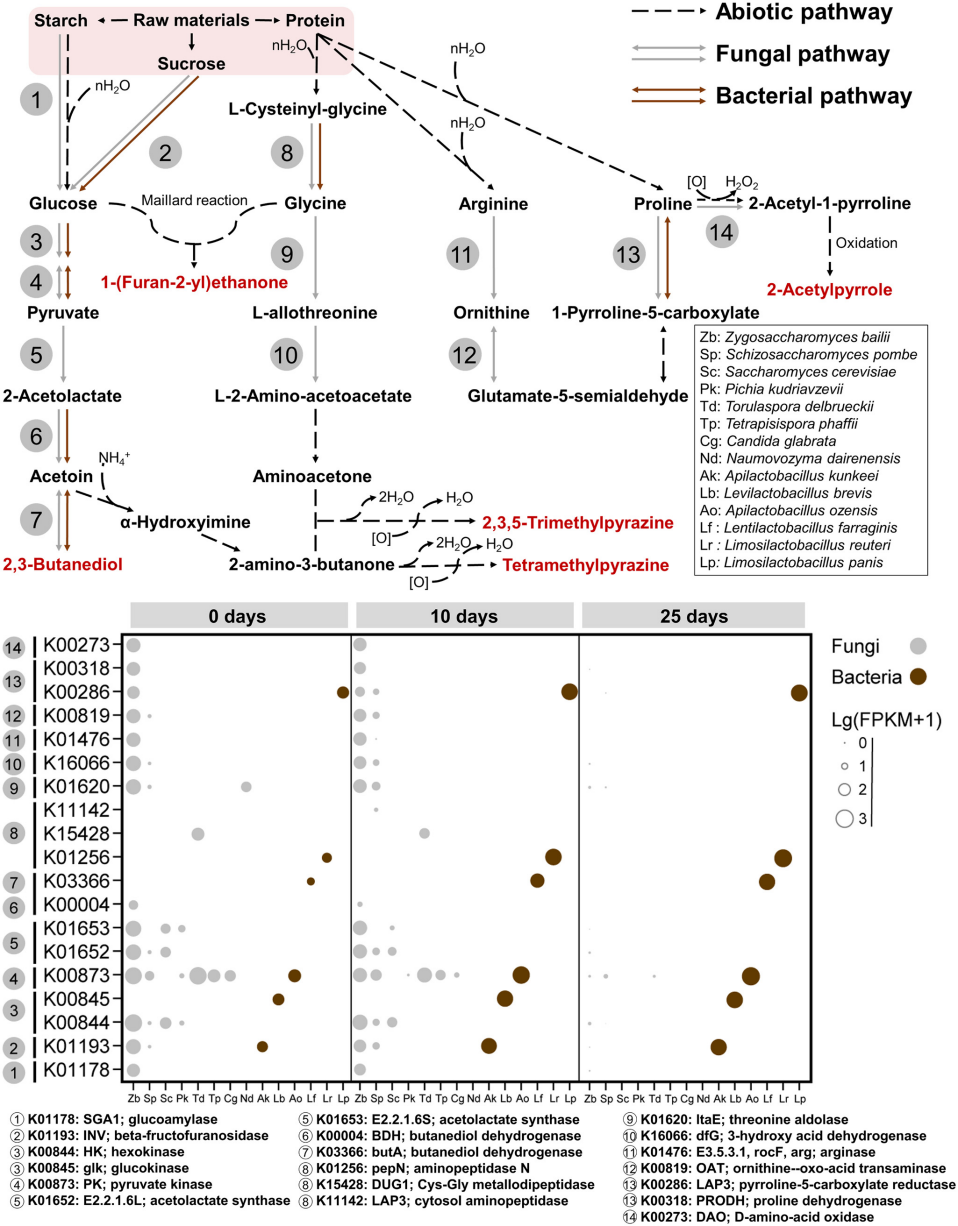
Synthetic pathway and molecular mechanism of representative chemical markers in the JX fermentation process. The pathway was constructed by mapping genes and chemicals to the KEGG database. The gene transcription levels were normalized to the FPKM values (detailed information is shown in Data Set S4 in the supplemental material).

Taking the JX group as an example, we identified the genes and pathways underlying partial fungus-bacterium metabolic interactions. We found that most community metabolic functions were dynamic interdependent interactions according to the fragments per kilobase of transcript per million fragments (FPKM) values of metabolism-related genes (Pearson’s correlation *r* > 0.6; *P < *0.01). Metabolism-related genes were involved mainly in functional categories of carbon, nitrogen, and sulfur metabolism during the fermentation process. During the early phase of fermentation, fungi like non-*Saccharomyces* yeasts were active in sulfur metabolism, terpenoid and polyketide metabolism, glyoxylate and dicarboxylate metabolism, lipid metabolism, and the tricarboxylic acid (TCA) cycle. During the medium and later phases, bacteria such as *Lactobacillus* were active in carbon metabolism, butanoate metabolism, propanoate metabolism, methane metabolism, galactose metabolism, nitrogen metabolism, and amino acid metabolism. We found that 7 core species were involved in the metabolism of 15 chemical markers or precursors of chemical markers in JX fermentation. Specifically, Zygosaccharomyces bailii contributed to the metabolism of 1,2-propanediol, benzoic acid, ethyl lactate, 2,3-butanediol, β-ethylphenethyl alcohol, and precursors of 2-acetylpyrrole, ethyl phenylacetate, ethyl nonanoate, and 2,3,5-trimethylpyrazine. Schizosaccharomyces pombe generated the precursors of 2,3-butanediol, 1-(furan-2-yl)ethenone, 1,2-propanediol, 2,3,5-trimethylpyrazine, ethyl lactate, ethyl phenylacetate, and ethyl nonanoate. Pichia kudriavzevii, Torulaspora delbrueckii, and Saccharomyces cerevisiae generated several organic acids and the precursors of 2,3-butanediol, 1-(furan-2-yl)ethenone, 1,2-propanediol, and β-ethylphenethyl alcohol. Limosilactobacillus panis and Limosilactobacillus reuteri generated the precursors of 1,2-propanediol, benzoic acid, ethyl lactate, 2,3-butanediol, and 2-acetylpyrrole. Based on the correlations between species and the corresponding chemical markers, we found abundant cometabolism interactions between fungi and bacteria across fermentation phases (Fig. S7 and Data Set S4). Representatively, both Zygosaccharomyces bailii and *Limosilactobacillus panis* contributed to the metabolism of 1,2-propanediol, benzoic acid, ethyl lactate, 2,3-butanediol, and 2-acetylpyrrole. Both *Pichia kudriavzevii* and *Limosilactobacillus reuteri* contributed to the metabolism of 2,3,5-trimethylpyrazine, ethyl lactate, and precursors of the Maillard reaction. Some cometabolism patterns, such as the interdependent interactions between Zygosaccharomyces and *Lactobacillus*, facilitated the synthesis of 1-(furan-2-yl)ethenone, 2-acetylpyrrole, 2,3-butanediol, 2,3,5-trimethylpyrazine, and tetramethylpyrazine (Fig. 5 and Fig. S6).

We then constructed the whole pathway from raw materials to these five chemical markers (Fig. 5). Overall, eight fungal genera (mainly yeasts) and six bacterial genera (mainly *Lactobacillus*) participated in the metabolism of chemical markers. Although yeasts and *Lactobacillus* coexisted during the entire fermentation process, the cometabolism process happened mainly in the early and medium phases. Specifically, during the first 10 days of fermentation, Zygosaccharomyces, Torulaspora delbrueckii, and other yeasts converted raw materials into glucose, acetoin, l-2-amino-acetoacetate, glutamate-5-semialdehyde, and other flavor precursors. Yeasts exhibited high FPKM values for SGA1 (glucoamylase), INV (beta-fructofuranosidase), BDH (butanediol dehydrogenase), dfG (3-hydroxy acid dehydrogenase), and OAT (ornithine-oxoacid transaminase). Next, *Lactobacillus*, along with abiotic reactions, converted these flavor precursors into chemical markers. We found the *Lactobacillus* exhibited high FPKM values for INV, butA (butanediol dehydrogenase), pepN (aminopeptidase N), and LAP3 (cytosol aminopeptidase) at 10 days of fermentation. After 25 days of fermentation, bacterial expression of INV, butA, pepN, and LAP3 gradually increased, while the metabolic functions of yeasts gradually diminished (Fig. 5).

### Biotic factors contribute to distinct baijiu microbiome assemblies.

**FIG 6:**
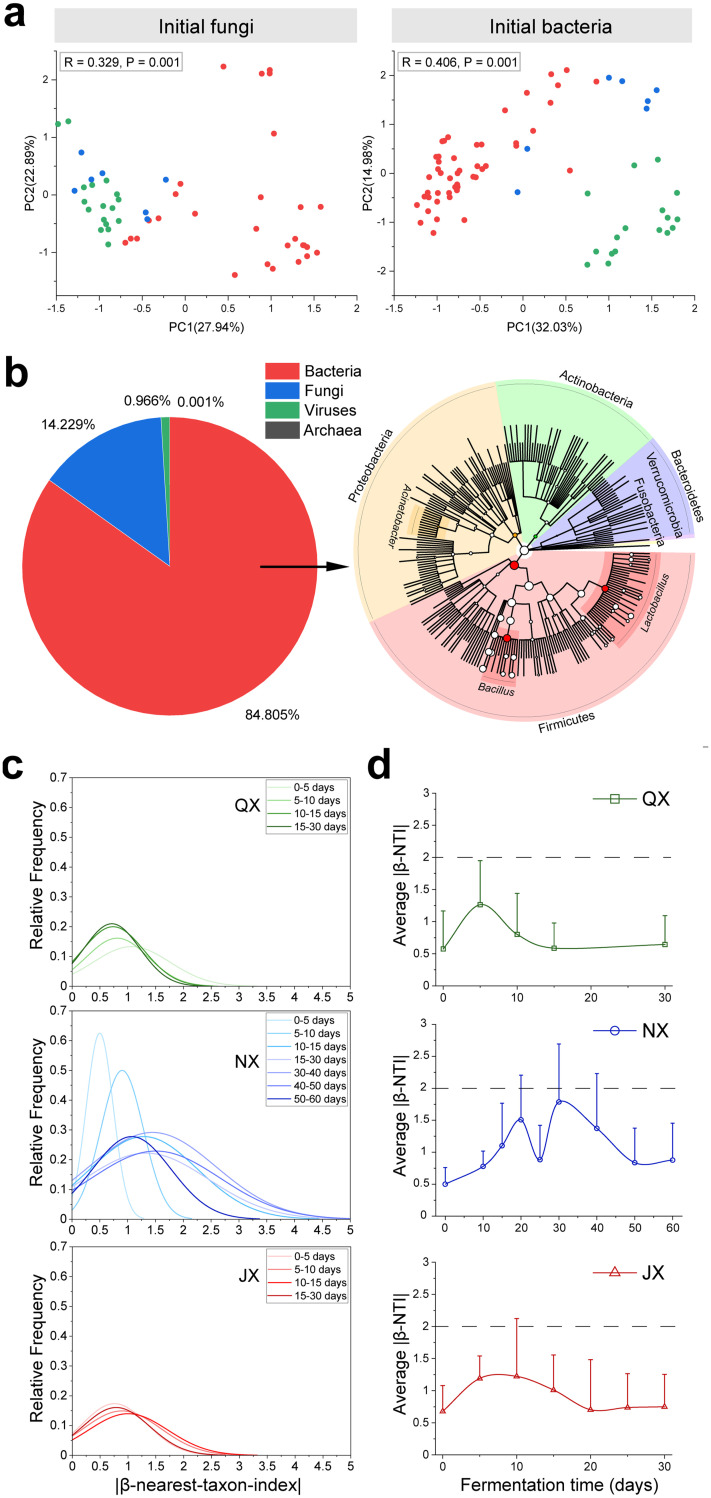
Different initial microbial structures and strain-level diversity contribute to distinct microbiome assemblies. (a) Principal-component analysis plot showing distinct initial fungal and bacterial structures among the three groups (green, QX [*n* = 17]; blue, NX [*n* = 7]; red, JX [*n* = 48]). (b) Composition of the total taxonomy annotation (left) and bacterial genomic diversity across six samples at the initial fermentation phase (right). (c) Distribution of beta nearest-taxon index (βNTI) values during the fermentation process. (d) Fermentation phases were identified by |βNTI| values. The observed value is from the mean of its associated null distribution. The initial phases were 0 to 5 days for QX, 0 to 20 days for NX, and 0 to 10 days for JX. The medium phases were 5 to 15 days for QX, 20 to 50 days for NX, and 10 to 20 days for JX. The later phases were 15 to 30 days for QX, 50 to 60 days for NX, and 20 to 30 days for JX.

We identified two biotic factors that can cause distinct baijiu microbiome assemblies by analyzing the initial biotic characteristics of the samples (Fig. 6). The two biotic factors are the initial microbial structure and microbial intraspecific diversity. By comparing the initial microbial structures of the three groups, we found that most core microbial genera were shared among the three groups but with different relative abundances, such as *Lactobacillus*, *Weissella*, Acinetobacter, *Oceanobacillus*, Pseudomonas, *Bacillus*, *Kroppenstedtia*, *Pediococcus*, *Acetobacter*, *Saccharomyces*, *Pichia*, *Wickerhamomyces*, and *Thermoascus*. The total relative abundance of the shared bacterial genera was 97.6%, and the total relative abundance of the shared fungal genera was 99.5%. Although the similarity of the microbial genera, we observed significant (*P* = 0.001 by ANOSIM) differences in the initial fungal and bacterial structures among the three groups (Fig. 6a). We found high initial relative abundances of Zygosaccharomyces, *Bacillus*, and *Kroppenstedtia* in the JX group; *Hyphopichia*, Aspergillus, *Pediococcus*, and *Weissella* in the QX group; and *Saccharopolyspora*, *Candida*, *Rhizopus*, and Pseudomonas in the NX group (Fig. S8). The high initial abundance of *Hyphopichia* enhanced its possibility of encountering other species in the QX group. As a result, *Hyphopichia* showed more interactions with bacteria in the QX group than in the NX and JX groups (Fig. 4). The high abundance of Zygosaccharomyces in the JX group and *Candida* in the NX group also showed similar effects on fungus-bacterium interactions. Different fungus-bacterium interactions could cause different microbial succession and assembly patterns. That microbial intraspecific diversity was another biotic factor that contributed to the microbiome assembly. Taking JX fermentation as an example, we found genomic diversity across fermentation samples with distinct species or strains of the same genera (Fig. 6b). Bacteria showed the highest values for both gene abundance (84.805%) and genomic diversity at the initial fermentation phase (Fig. 6b and Data Set S5). *Lactobacillus*, *Bacillus*, and Acinetobacter were the three core microbial genera that showed the most diverse strain-level or species-level diversity, suggesting that some of the same core bacterial genera might have different interactions with fungi (Fig. 4 and 5) or different functional traits (Data Set S6). Variable fungus-bacterium interactions at the genus taxonomic level could also cause fluctuations in the microbiome assembly pattern.

We then used the beta nearest-taxon index (βNTI) (null models to assess whether the total microbial variation was higher than the predicted value) to show the effect of biotic factors on the assembly patterns (Fig. 6c). We found that all three groups underwent a three-phase assembly process, from a stochastic assembly process followed by an almost determined assembly process (the total microbial variation of most samples were lower than the predicted values) to a stochastic assembly process. The contribution of biotic factors to distinct stochastic microbiome assemblies began early during fermentation (Fig. 6c). For the QX group, the community assembly shifted from stochastic to an almost determined phase between day 5 and day 10 of fermentation. During this phase, the average |βNTI| value (absolute value of beta-NTI) of the QX group was approximately 1.1, and the maximum βNTI value was approximately 3.5, indicating an almost determined assembly process. For the JX group, the community assembly experienced a phase shift between day 10 and day 15 of fermentation. During this phase, the maximum |βNTI| value for the JX group was approximately 3.3, and the average |βNTI| value was approximately 1.2, indicating an almost determined assembly process. For the NX group, the community assembly underwent a phase shift between day 20 and day 40 of fermentation. The average |βNTI| value was approximately 1.46 in the NX group, and the maximum |βNTI| value was approximately 6.1 during the shift phase (Fig. 6d).

### Regulation of microbial flavor metabolism in simulated fermentation.

**FIG 7:**
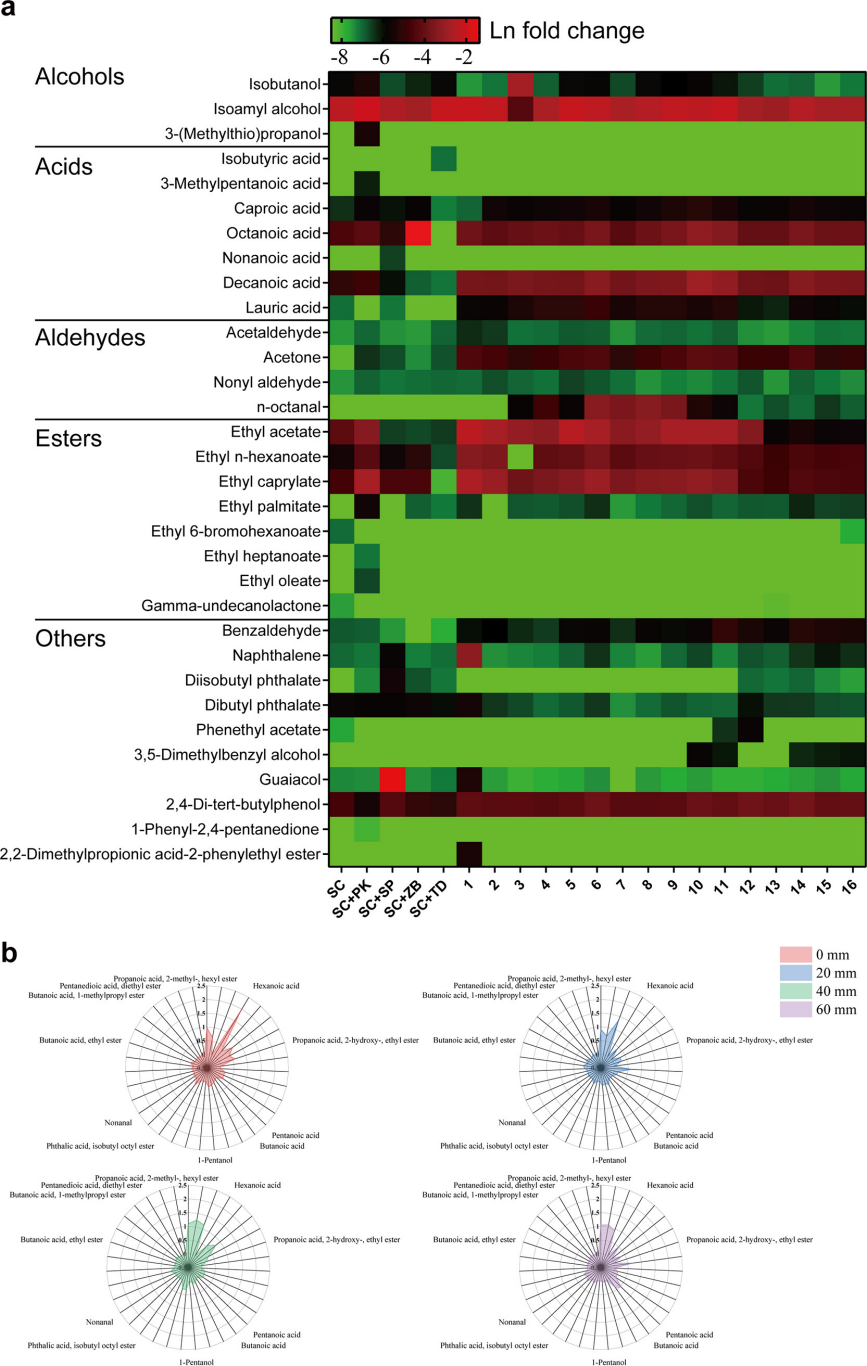
Core yeast composition and microbial distribution trigger distinct metabolic profiles in simulated fermentations. (a) Volatile profiles (parts per million) at the end of 3 days of fermentation. The combination ratios of PK to SC to SP to ZB to TD were 1:1:1:1:1 (1), 1:2:2:2:2 (2), 1:3:3:3:3 (3), 1:4:4:4:4 (4), 2:1:2:3:4 (5), 2:2:1:4:3 (6), 2:3:4:1:2 (7), 2:4:3:2:1 (8), 3:1:3:4:1 (9), 3:2:4:3:1 (10), 3:3:1:2:4 (11), 3:4:2:1:3 (12), 4:1:4:2:3 (13), 4:2:3:1:4 (14), 4:3:2:4:1 (15), and 4:4:1:3:2 (16). (b) Metabolic profiles (parts per million) of fermentations with different initial distances of the inoculated strains.

We created and validated two optional approaches for regulating microbial flavor metabolism based on the characteristics of the geographical baijiu microbiome assembly. First, Fig. 7a shows the different concentrations of flavor-related chemicals regulated by different initial microbial abundances. The five-strain communities generated significantly higher concentrations of acids, esters, and aromatic chemicals than did the one- or two-strain communities. Specifically, combinations 1 and 2 generated the highest concentrations of esters, which were 7.317 and 5.206 times higher than those with Saccharomyces cerevisiae, respectively. Combinations 10 and 11 generated the highest concentrations of acids, which were 5.371 and 4.385 times higher than those with Saccharomyces cerevisiae, respectively. Combinations 6 and 8 generated the highest concentrations of aromatic chemicals, which were 5.415 and 4.488 times higher than those with Saccharomyces cerevisiae, respectively. We found that combination 11 (PK (*Pichia kudriavzevii*)/SC (*Saccharomyces cerevisiae*)/SP (*Schizosaccharomyces pombe*)/ZB (*Zygosaccharomyces bailii*)/TD (*Torulaspora delbrueckii*) ratio of 3:3:1:2:4) showed the highest accumulated concentrations of flavor-related chemicals and was regarded as the most efficient yeast combination of the simulated fermentation. Second, Fig. 7b shows the different metabolic profiles generated by the same microbial strains by regulating the initial spatial distributions of Saccharomyces cerevisiae and Fructilactobacillus fructivorans (Fig. S9). The concentrations of nonanal, ethyl 2-hydroxypropionate, 1-pentanol, hexanoic acid, isobutyl phthalate, and ethyl butyrate all decreased with increasing strain distance.

## DISCUSSION

### Geographical characteristics of fermented chemical compositions.

Determining the flavor-related chemical markers of baijiu is an ever-challenging target for academic and industrial research. Marker chemical compositions are essential for identifying different geographical characteristics of flavor and fermentation quality (6, 28–30). Our work illustrated 20 to 40 chemical markers out of 471 volatile chemicals for each basic baijiu aroma type (Table 1). The number of markers chemicals agrees with the characteristic ratios (about 3 to 40 genuine key odorants) of food flavor (6). The identified chemical markers include both key odorants and quality risk chemicals such as *p*-cresol (31). However, the results may not fully include all genuine key odorant chemicals due to errors from the statistical model. For example, some key odorant chemicals (32) with low concentrations showed low VIP values in the statistical model, whereas some noncritical odorant chemicals with high sample coverage showed high VIP values in the statistical model (see Table S2 in the supplemental material). Future work combining quantification and sensory approaches (33) to evaluate these chemical markers would help us not only further determine the quality of fermentation flavor but also assess the quality risks of production. For instance, ethyl ester and β-damascenone were previously reported to be key flavor-related chemicals for the formation of QX aroma (34). Some chemicals such as ethyl caproate, ethyl 3-phenylpropanoate, hexanoic acid, and *p*-cresol have been reported for their sensory characteristics in NX fermentation (33, 35). Some chemicals such as 3-methylbutanoic acid and tetramethylpyrazine were previously reported to be flavor-related chemicals in the JX group (22).

### Geographical characteristics of the baijiu microbiome.

Microbial resources are the most important members in flavor biotechnological processes. Our work revealed that the baijiu microbiome consists of about 735 bacterial genera and 290 fungal genera (Data Set S4). In JX fermentation, the baijiu microbiome was active in the expression of over 32,373 genes (Data Set S6). Compared with less complex fermentations (7), the baijiu microbiome provides more abundant microbial resources for generating almost all kinds of flavor-related volatile chemicals containing less than 20 carbon atoms (36).

For most spontaneous fermentations, fermentation microbiomes are usually artificially isolated or enriched from the local environmental microbial pools (19, 37–39) that often have different microbial diversity and metabolic functions in latitude gradients (40). Similar to the geographical characteristics of the wine microbiome (4), our work revealed that fungal genera rather than bacterial genera showed significant differences during fermentation among different latitude groups (Fig. 2). Notably, these results do not mean that bacterial diversity is the same among different areas. Future work using metagenome sequencing on a large scale could help us better understand microbial geographical characteristics at a deeper taxonomic level. Nevertheless, we confirm that the baijiu microbiome is flavor related and geography dependent.

The geographical characteristics of the baijiu microbiome could be ascribed to the enrichment of species from various microbial pools (41). At a large scale, the local microbial pool is largely governed by pH, precipitation, and nutrition (4, 40), suggesting that different areas may possess the corresponding suitable natural conditions to produce one typical baijiu aroma type. With climate change, the functional complexity and diversity of local microbial pools are severely impacted (42). The predominant environmental microbiome is essential for flavor quality in spontaneous fermentations (19). Such microbial pool changes may cause quality fluctuations in geography-dependent flavor without environmental microbiome control. Our work provided a representative sink for source tracking (41, 43) in each geography-dependent flavor group (Fig. 2). It would be fascinating to see how to regulate the fermentation environment to face global climate change. In addition, reproducing the desired food flavor regardless of the geographical conditions of the product’s origin would be possible.

### Interactions between geographical chemical markers and the baijiu microbiome.

The changeable strength of microbial interactions could affect the efficiency of chemical marker metabolism (11). Our results predicted abundant fungus-bacterium interactions that contribute to chemical marker metabolism (Fig. 4). Although the baijiu microbiome correlated with almost all volatile chemicals, the variance explained is limited (Fig. 3b), suggesting the potential importance of abiotic reactions (Maillard reactions and cereal enzymes, etc.) (44) or biomass-independent correlations (high-order interactions, etc.) (45, 46). Within the predictable range, fungus-bacterium interactions account for most of the explained variance. Thus, we confirm that a higher correlation of fungus-bacterium metabolic interactions facilitates the metabolism of flavor-related chemicals.

As for the interactions that we precited for the JX network, a recent study revealed that the interaction between *Pichia* and *Monascus* is driven by the biosynthesis of 2-phenylethanol. Volatile flavor chemical markers such as 2-phenylethanol will inhibit conidium germination and mycelial growth by filamentous fungi (47). Notably, the generation of 2-phenylethanol can also be achieved by metabolic cooperation between Zygosaccharomyces bailii and Levilactobacillus brevis (Fig. S6). The mechanisms of many microbial interactions through volatile chemicals in the network (Fig. 4) are still awaiting exploration.

### Fungus-bacterium dynamic cometabolism of geographical chemical markers.

Although predicted associations between the microbiome and chemical markers have the enormous potential to reproduce fermentation flavor, the yields of flavor-related chemicals are usually too low for commercial applications (48). We cannot efficiently obtain the desired flavor production without understanding the metabolic pathways of flavor-related chemicals. Taking JX fermentation as an example, we constructed whole biotic and abiotic reactions of heterocyclic chemical markers (Fig. 5 and Data Set S4). The constructed pathway provided a blueprint for the design of engineered microbiomes to produce flavor-related chemicals. Notably, partial steps in the constructed pathway also relied on chemical conversions and enzymes (49, 50). Understanding the contribution of these abiotic factors is also important and awaits further research in the future. With the further optimization of the microbiome based on the design-build-test-learn cycle, we will be able to efficiently reproduce fermentation flavors for commercial applications (27). In addition, some flavor-related chemicals such as 2-acetylpyrrole may also be efficiently produced for biofuel use.

### Biotic factors contribute to distinct geographical baijiu microbiome assemblies.

Understanding microbiome assemblies is the first prerequisite for ensuring reproducible fermentation outcomes because different microbiome assembly patterns will cause different functional outputs (51). We found that the baijiu microbiome underwent different three-phase ecological processes among the three groups (Fig. 6c). Microbiome assembly is driven by both deterministic factors (temperature, organic acids, and ethanol) (17, 36, 52) and stochastic processes. Deterministic factors can constrain microbial functional diversity (53), suggesting that the microbiome assembly will affect flavor formation at different time points of ecological process shifts. Food fermentation usually ends with the convergence of microbial compositions (2, 12, 13). Thus, we believe that the different time points of phase shifts could be the reason why different baijiu aroma types undergo fermentation for different times. The balance between the speed of microbial fermentation within phases and the speed of phase shifts would be another challenge to solve for efficient and reproducible high-complexity fermentations.

Notably, studies of less complex fermentations demonstrated that unstable microbiome assemblies and functional outputs are ascribed largely to intraspecific diversity and phage infections (12, 16). Here, although the existence of microbial phages or viruses was detected (Fig. 6b), the effect of infection on baijiu fermentation is not clear.

### Regulation of microbial flavor metabolism in simulated fermentations.

In many food fermentations, the addition of starters (koji, etc.) that are related to the initial microbial inoculum for fermentation is the key step in the regulation of fermentation flavor (1). Here, we validated that we could regulate fermentation flavor by adjusting the initial microbial combination or the initial microbial spatial distribution (Fig. 7). These two approaches can help optimize the design of synthetic fungus-bacterium communities in a new round of the design-build-test-learn cycle (27). Collectively, we confirm that reproducing the desired fermented flavor can be achieved with a well-designed fungus-bacterium cofermentation system.

## MATERIALS AND METHODS

### Fermentation.

Chinese liquor has three typical aroma types, named Qingxiang (QX) (light-aroma type), Nongxiang (NX) (strong-aroma type), and Jiangxiang (JX) (sauce aroma type). The spontaneous solid-state fermentation of Chinese liquor is done in sealed pits (a sort of fermentation chamber) and lasts approximately 30 days for QX and JX and 60 days for NX (17, 18, 36).

### Sampling.

To assess the geographical characteristics of the chemical and microbial compositions, we collected 403 fermented samples, including 101 samples of QX, 98 samples of NX, and 204 samples of JX, from 9 representative distilleries in China (Fig. 1a; see also Table S1 in the supplemental material). The collected samples covered five to six batches of fermentation within each group. To keep the uniformity of sampling, samples were taken at selected intervals of time and space during fermentation. Specifically, samples were taken at intervals of about 5 days during fermentation. At each sampling time point, samples were taken at the same depth (0.5 m and 1.0 m) of each pit. All collected samples were transferred to a bucket filled with dry ice and transported to the laboratory within 24 h. We separated consistent 500 g of fermented grains from each sample for further analyses of volatile compounds and microbial characteristics.

### Volatile compound analysis.

Volatile chemicals of samples were identified by headspace solid-phase microextraction–gas chromatography–mass spectrometry (HS-SPME-GC-MS) as described previously (54). All samples were pretreated with 20 mL of sterile saline (1% CaCl_2_, 0.85% NaCl) in 50-mL centrifuge tubes to collect the supernatants after centrifugation at 5,000 × *g* for 10 min. The supernatant (8 mL) was added to headspace bottles and mixed with 3 g of NaCl and an internal standard (10 μL menthol).

### DNA and RNA extraction.

To reveal why microbial geographical diversity can contribute to the formation of distinct flavors, we extracted the total DNA and RNA from samples for further sequencing and bioinformatic analysis. The samples were pretreated with sterile phosphate-buffered saline (PBS) (0.1 mol/L, pH 7.2 to 7.4) and centrifuged at 300 × *g* for 5 min. Next, the supernatants were centrifuged at 11,000 × *g* for 5 min to obtain sediments.

For DNA extraction, the sediments were cooled, milled in liquid nitrogen, and extracted using sodium laurate buffer (sodium laurate at 10 g/L, Tris-HCl at 0.1 mol/L, NaCl at 0.1 mol/L, and EDTA at 0.02 mol/L) with phenol-chloroform-isoamyl alcohol (25:24:1) to obtain total DNA. The quality of the total DNA was assessed by 1% agarose gel electrophoresis with a NanoDrop 8000 spectrophotometer (Thermo Scientific, Waltham, MA) (260-nm/280-nm ratio). All genomic DNA of the samples was stored at −80°C for further procedures.

For RNA extraction, the sediments were milled with liquid nitrogen, and total RNA was extracted with sodium laurate buffer (sodium laurate at 10 g/L, Tris-HCl at 0.1 mol/L, NaCl at 0.1 mol/L, and EDTA at 0.02 mol/L) containing TRIzol (Sigma-Aldrich, St. Louis, MO). A Ribo-Zero rRNA removal kit (bacteria) and a Ribo-Zero magnetic gold kit (yeast) (Epicentre, San Diego, CA) were used to remove rRNA from the total RNA. The RNA of the samples was then stored at −80°C for further procedures.

### DNA and RNA sequencing.

For DNA sequencing of marker genes, the V3-V4 hypervariable region of the 16S rRNA gene and the internal transcribed spacer 1 (ITS1)/ITS2 region were PCR amplified as previously described (36, 54). The resulting amplicons were quantified and sequenced on the Illumina MiSeq PE300 sequencing platform (Illumina, San Diego, CA), which was conducted by the Allwegene Technology Company (Beijing, China). Low-quality samples were removed before bioinformatic analysis.

For DNA sequencing of the metagenome, the genomic DNA was randomly broken into fragments with a length of about 350 bp by a sonicator. Next, the whole library was prepared by steps of terminal repair, A-tail addition, ligation of the adaptors to the fragments, purification, and PCR amplification. Illumina (San Diego, CA) HiSeq sequencing was performed after qualified library pooling.

For RNA sequencing, metatranscriptomic libraries were constructed according to the instructions of the NEBNext Ultra RNA library prep kit (Illumina) (New England BioLabs, Ipswich, MA) and sequenced on the Illumina (San Diego, CA) HiSeq 2500 platform, which was conducted by the Allwegene Technology Company (Beijing, China).

### Bioinformatic analysis.

For raw DNA sequencing reads, we trimmed low-quality sequences according to the average *Q*_20_ quality standard (55). Next, overlapping reads were merged by fastq-join, primer sequences were removed, and only completely assembled reads were used for further analysis. The overlap length of merging was set to be no less than 20 bp, and the minimum length of fungal sequences was set at 50 bp. The unique sequence set was classified into amplicon sequence variants (ASVs) via QIIME 2 (version 2019-04) under default thresholds. Chimeric sequences were identified and removed using DADA2 (55). The bacterial ASVs were mapped to the SILVA 132 database for taxonomic identification. The annotated *Lactobacillus* ASVs in this study represented the sum of *Lactobacillus*, *Lacticaseibacillus*, *Lactiplantibacillus*, *Latilactobacillus*, *Liquorilactobacillus*, *Levilactobacillus*, *Lentilactobacillus*, *Loigolactobacillus*, *Limosilactobacillus*, *Fructilactobacillus*, *Companilactobacillus*, *Acetilactobacillus*, and *Apilactobacillus* based on the SILVA 138 database annotation. The fungal ASVs were mapped to the Unite database (version 8.2) for taxonomic identification.

For raw metagenomic DNA sequencing fragments, the raw data were filtered to obtain clean data based on the *Q*_20_ value. Clean reads were assembled using the MEGAHIT (v1.0.6) (56) assembly program (–min-count 2 –k-min 27 –k-max 87 –k-step 10). Contigs with a length of less than 500 bp were filtered. Metabat2 was used to perform the binning process based on contigs. The metagenome-assembled genomes (MAGs) were assessed by Checkm. Open reading frame prediction was performed using PRODIGAL (57) and filtered using a length of <100 nucleotides (nt). CD-HIT (58) (with set -c 0.95, -G 0, -aS 0.9, -g 1, -d 0) was used to remove redundancy from the predicted gene sequences. The reads of each gene in different samples was calculated using Bowtie (59) and normalized to obtain a gene abundance table.

For RNA sequences, we then performed species classification analysis, species complexity analysis, as well as gene expression abundance analysis. We compared the high-quality reads to the reads in the nonredundant (Nr) protein, metabolic pathway (KEGG), gene ontology (GO), protein family (Pfam), homologous gene cluster (eggNOG), and carbohydrate-active enzyme (CAZy) databases to obtain functional annotation information. The sequencing reads for each sample were remapped to the reference sequences using RSEM software (60). Gene expression levels were measured using the fragments per kilobase of transcript per million fragments (FPKM) method based on the number of uniquely mapped reads (61). The DESeq package (version 2.1.0) was employed to detect differentially expressed genes (DEGs) between two groups (62). The false discovery rate (FDR) was applied to correct the *P* value threshold in multiple tests (63). An FDR-adjusted *P* value (*q* value) of ≤0.05 and a |log_2_ fold change| of >1 were used as the thresholds for identifying significant differences in gene expression in this study.

### Network analysis of fungus-bacterium and microbiota-metabolite interactions.

To assess major microbial contributors of flavor-related metabolites, molecular ecological network analysis was applied with online tools using default parameters (64). All interaction thresholds were calculated according to random matrix theory to filter node associations. Core fungal and bacterial genus interactions were filtered out based on 20% coverage of samples in each group.

### Calculation of ecological processes.

The beta nearest-taxon index (βNTI) was calculated according to a protocol described previously (65). We excluded some low-confidence (average relative abundance of <0.008%) representative microbial sequences due to the difficulty in calculating the large microbial evolutionary tree. βNTI values of less than −2 or greater than +2 indicate a statistically significant divergence between the observed and expected beta mean nearest-taxon distances. Generally, a higher |βNTI| value represents fewer stochastic effects (65).

### Isolation and identification of microorganisms.

For microbial species that were used in simulated fermentations, five yeasts and *Fructilactobacillus fructivorans* were isolated from pooled fermented samples of JX. To isolate microbial strains, 50 g of fermented samples was homogenized in 250 mL of sterile PBS in a shaking incubator at 200 rpm for 30 min at 30°C. Next, 1.0 mL of the homogenate was diluted 10-fold in a sterile saline solution (0.9% [wt/vol] NaCl). Using methods similar to the ones described in our previous study (52), we spread 100 μL of the diluted homogenate onto the surface of Luria-Bertani (LB) agar and potato dextrose agar (PDA) plates. PDA plates were used to isolate fungi and were incubated at 30°C for 48 h. LB agar plates were used to isolate bacteria and were incubated at 37°C for 48 h. We used the universal primer set 27F (5′-AGA GTT TGA TCM TGG CTC AG-3′) and 1492R (5′-CGG TTA CCT TGT TAC GAC TT-3′) to amplify the 16S rRNA genes of bacteria. The ITS region of fungi was amplified with primers ITS1 (5′-TCC GTA GGT GAA CCT GCG G-3′) and ITS4 (5′-TCC TCC GCT TAT TGA TAT GC-3′). The PCR products were sequenced by a pipeline created by Genewiz (Suzhou, China). Next, we subjected the sequences to a BLAST search against the NCBI database, and the results were used to identify the isolates. Because the isolated species were not type strains, we just named the isolated organisms Saccharomyces cerevisiae, *Pichia kudriavzevii*, Zygosaccharomyces bailii, Schizosaccharomyces pombe, Torulaspora delbrueckii, and *Fructilactobacillus fructivorans* according to the BLAST results.

### Regulation of the microbial generation of flavor-related chemicals in simulated fermentations.

We created and validated two optional approaches for the regulation of the microbial generation of flavor-related chemicals based on the geographical characteristics of baijiu fermentation. The first simulated fermentation aimed to validate that different initial microbial structures can generate different flavor-related chemical profiles. If we have different demands for flavor-related chemicals, the best microbial combination design could be different even with the same microbial members. We established 21 synthetic microbial communities of one yeast, two yeasts, or five yeasts (Table S5) to compare the metabolic profiles after 3 days of fermentation. The simulated still fermentations were conducted at 30°C with the same initial biomass (2 × 10^6^ CFU) in liquid medium containing sorghum extract. Each simulated community had three replications of fermentation. The volatile compounds of fermented samples were analyzed by GC-MS using the same process as the one described above. Next, we screened out the most efficient yeast combinations according to the total concentration of flavor-related chemicals.

We assumed that different abundances of strains (yeasts, etc.) in solid fermentation material would provide different possible opportunities to interact with other strains. The speed and possibility of fungus-bacterium interactions may impact microbial flavor metabolism. Thus, the second simulated fermentation aimed to validate that different speeds and possibilities of fungus-bacterium interactions could affect fungus-bacterium cometabolism and, thus, flavor formation in solid-state fermentations. We designed a fungus-bacterium fermentation system under four cell spatial distances (0 mm, 2 mm, 4 mm, and 6 mm) to create different microbial interaction possibilities. Saccharomyces cerevisiae and *Fructilactobacillus fructivorans* were isolated from JX fermentation samples as flavor producers. We inoculated the two strains into a solid fermentation matrix (sorghum extract medium with agar added) with the same biomass (2 × 10^6^ CFU) at the start of fermentation (Fig. S9). After 10 days of simulated fermentations, we harvested and smashed the fermented material to conduct further volatile compound analysis.

### Statistical analysis.

The dynamics of physical and chemical factors were fitted with OriginPro2019. Principal-component analysis, two-way orthogonal partial least-squares modeling, and variable importance in projection analysis were done via SIMCA-P (version 13.0). Mantel tests, ANOVA, ANOSIM, microbial correlation analysis, canonical correspondence analysis, and virtual population analysis were carried out using the vegan package in R (http://vegan.r-forge.r-project.org/). *P* values were adjusted for nonparametric analysis using the Statistical Package for the Social Sciences (SPSS) (version 22).

### Data visualization.

Data were plotted in OriginPro2019, Microsoft Excel, and Adobe Illustrator CS6. The network was visualized via Gephi (version 0.9.2).

### Data availability.

The reads of marker genes sequencing can be accessed in the PRJNA377357, PRJNA396629, and PRJNA837950. The reads of metatranscription sequencing can be accessed in the PRJNA837634. The reads of metagenome sequencing can be accessed in the PRJNA837610.
