# Supplementary material for: Geographically Associated Fungus-Bacterium Interactions Contribute to the Formation of Geography-Dependent Flavor during High-Complexity Spontaneous Fermentation
Source: Microbiol Spectr. 2022 Sep 22;10(5):e01844-22. doi: 10.1128/spectrum.01844-22 (PMC9603688; doi:10.1128/spectrum.01844-22)

Supplementary materials

Geographically associated fungi-bacteria interactions contribute to the formation of geography-dependent flavor during high-complexity spontaneous fermentation

Yuwei Tan[a, b, 1], Hai Du[a,1], Hongxia Zhang[a], Chen Fang[a], Guangyuan Jin[a], Shuang Chen[a], Qun Wu[a], Yan Zhang[c], Menghui Zhang[d, *], Yan Xu[a, *]

[a] Laboratory of Brewing Microbiology and Applied Enzymology, School of Biotechnology and Key Laboratory of Industrial Biotechnology of Ministry of Education, Jiangnan University, Wuxi, Jiangsu, China

[b] Bioprocess Engineering, Wageningen University and Research, P.O. Box 16, 6700AA Wageningen, Netherlands

[c] Key Laboratory of Systems Biomedicine (Ministry of Education), Shanghai Center for Systems Biomedicine, Shanghai Jiao Tong University, Shanghai 200240, China

[d] State Key Laboratory of Microbial Metabolism and Joint International Research Laboratory of Metabolic and Developmental Sciences, School of Life Sciences and Biotechnology, Shanghai Jiao Tong University, Shanghai 200240, China

1. Both authors contributed equally

* Corresponding authors, E-mail address: yxu@jiangnan.edu.cn

This supplementary file includes:

Supplementary figures: Fig. S1–Fig. S9

Supplementary tables: Table S1–Table S5

Other supplementary information for this manuscript includes:

Supplementary dataset.xlsx: Dataset 1–Dataset 6

Dataset 1 Volatile compounds in fermented grains detected by HS-SPME-GC-MS.

Dataset 2 Relative abundance of dominant fungal and bacterial genera among three groups.

Dataset 3 Significant microbial interactions and microbiota-chemical interactions.

Dataset 4 Microbial gene transcription during JX fermentation process.

Dataset 5 Merged abundance table of *baijiu* microbiome based on MAGs.

Dataset 6 Normalized abundance of accessory genes based on NCBI database.

Supplementary figures caption:

Figure S1| a, The Venn diagram of volatile chemicals in the three aroma-type fermented grains identified via HS-SPME-GC-MS detection. **b,** Relative concentration of chemicals in the end of fermentation.

Figure S2| The heatmap of the volatile chemical markers based on relative concentration in fermented grains among three groups.

Figure S3| Venn plot of bacterial and fungal diversity at genera taxonomic level.

Figure S4| The microbial NMDS1, NMDS2, NMDS3 is significantly linked to the geography latitude. The lines represent the regression line fitted by the first-order polynomial.

Figure S5| Detailed significant associations between chemical markers and core microbial community at **a,** phylum and **b,** genus taxonomic level. The color of edges in networks are same as chemical nodes. The thickness of edges represents correlation values between nodes.

Figure S6| Fungi-bacteria metabolic interactions contribute to the generation of chemical markers in JX group. **a,** cross-feeding between *Zygosaccharomyces bailli* and *Lactobacillus panis*. **b,** *Zygosaccharomyces bailli* generate phenyl-acetaldehyde that facilitating the metabolism of phenyl-ethylalcohol by *Lactobacillus brevis*. **c,** cooperative metabolism of pyrazines by *Zygosaccharomyces bailli* and *Lactobacillus farraginis*.

Figure S7| KEGG functions of microbial community during the initial, medium and later fermentation phases. The heatmap values were normalized to FPKM (detailed information is shown in Dataset 4).

Figure S8| Heatmap analysis of initial microbial structure based on relative abundance.

Figure S9| Simulated fermentation with *Saccharomyces cerevisiae* and *Lactobacillus fructivorans* under different strains distance.

Supplementary tables

Table S1. Sampling Information

Group ID	Valley	Absolute latitude (°N)	Absolute longitude (°E)	Aroma type	Sampling size
LAOBAIGAN	the Yellow River	37.82	115.71	QX	53
FENJIU	the Yellow River	37.33	111.90	QX	12
QINGKE	the Yellow River	36.92	101.92	QX	36
JIANNANCHUN	Yangtze River	31.34	104.19	NX	29
WULIANGYE	Yangtze River	28.58	104.92	NX	33
XIJIU	Chishui River	28.33	106.19	NX	36
XIJIU	Chishui River	28.33	106.19	JX	40
MOUTAI	Chishui River	27.85	106.37	JX	144
GUOTAI	Chishui River	27.86	106.37	JX	20

Table S2. VIP values of volatile chemicals based on PLS model among three groups

Var ID (CAS number)	Var ID (Volatile compounds)	M1.VIP values
000505-10-2	3-methylthiopropanol	2.635
000123-66-0	Ethyl caproate	2.383
000078-83-1	Isobutanol	2.271
002548-87-0	(E)-2-octenal	2.264
000066-25-1	Hexanal	2.218
000106-44-5	p-Cresol	2.059
000098-86-2	Acetophenone	2.016
000123-51-3	3-Methyl-1-butanol	2.002
000100-51-6	Benzyl alcohol	1.987
000539-82-2	Ethyl valerate	1.980
000108-64-5	ethyl 3-methylbutanoate	1.979

001072-83-9	2-Acetyl pyrrole	1.964
000513-85-9	2,3-Butanediol	1.945
000111-14-8	Heptanoic acid	1.941
000124-19-6	1-Nonanal	1.930
000106-30-9	Ethyl heptanoate	1.914
001192-62-7	1-(Furan-2-yl)ethanone	1.903
000503-74-2	Butanoic acid, 3-methyl-	1.902
000124-07-2	Octanoic acid	1.880
000105-54-4	Ethyl butyrate	1.800
000079-31-2	Isobutyric acid	1.789
003777-69-3	Furan, 2-pentyl-	1.787
000142-92-7	Acetic acid, hexyl ester	1.757
001124-11-4	Tetramethylpyrazine	1.715
000057-55-6	1,2-Propanediol	1.705
014667-55-1	2,3,5-Trimethylpyrazine	1.685
000124-06-1	Ethyl myristate	1.638
033442-47-6	beta.-Ethylphenethyl alcohol	1.637
000098-00-0	Furfuryl alcohol	1.614
000091-20-3	Naphthalene	1.602
000060-12-8	Phenethyl alcohol	1.571
001191-41-9	9,12,15-Octadecatrienoic acid, ethyles	1.570
002198-61-0	Isopentyl hexanoate	1.563
000111-61-5	Octadecanoic acid, ethyl ester	1.513
023726-93-4	2-Buten-1-one, 1-(2,6,6-trimethyl-1,3-c	1.508
000065-85-0	Benzoic acid	1.483
001193-79-9	2-Acetyl-5-methylfuran	1.432
000106-32-1	Ethyl caprylate	1.423
055554-09-1	Tetradecanoic acid, 2-methyl-, methyl ester	1.421
000106-33-2	Ethyl laurate	1.418
000107-92-6	Butanoic acid	1.415

000101-97-3	Ethyl phenylacetate	1.405
000142-60-9	Propanoic acid, octyl ester	1.394
000142-62-1	Hexanoic acid	1.391
000540-07-8	Hexanoic acid, pentyl ester	1.379
006378-65-0	Hexanoic acid, hexyl ester	1.378
019329-89-6	Isoamyl lactate	1.376
014010-23-2	Heptadecanoic acid, ethyl ester	1.360
000075-07-0	acetaldehyde	1.351
005779-95-3	Benzaldehyde, 3,5-dimethyl-	1.348
000626-82-4	Hexanoic acid, butyl ester	1.335
000123-29-5	Nonanoic acid, ethyl ester	1.326
000105-79-3	Hexanoic acid, 2-methylpropyl ester	1.323
002021-28-5	Ethyl 3-phenylpropionate	1.309
000123-25-1	Diethyl succinate	1.283
006314-97-2	Benzene, (2,2-diethoxyethyl)-	1.281
000122-78-1	Benzeneacetaldehyde	1.267
000626-77-7	Propyl caproate	1.249
000079-09-4	Propanoic acid	1.240
000109-25-1	Heptanoic acid, 3-methylbutyl ester	1.228
002035-99-6	Octanoic acid, 3-methylbutyl ester	1.200
007786-61-0	2-Methoxy-4-vinylphenol	1.183
000496-16-2	Benzofuran, 2,3-dihydro-	1.153
041114-00-5	Pentadecanoic acid, ethyl ester	1.143
001797-74-6	Benzeneacetic acid, 2-propenyl ester	1.136
001117-55-1	Octanoic acid, hexyl ester	1.133
000589-98-0	3-Octanol	1.116
000111-62-6	Ethyl oleate	1.106
013327-56-5	Ethyl 3-methylthiopropionate	1.104
005973-71-7	Benzaldehyde, 3,4-dimethyl-	1.094
000128-37-0	Butylated Hydroxytoluene	1.092

000544-35-4	Linoleic acid ethyl ester	1.074
000097-64-3	Ethyl lactate	1.071
000628-97-7	Ethyl palmitate	1.069
000100-42-5	Styrene	1.060
000123-07-9	4-Ethylphenol	1.058
024683-00-9	Pyrazine, 2-methoxy-3-(2-methylpropyl)-	1.057
000143-08-8	1-Nonanol	1.023
000093-89-0	Ethyl benzoate	1.019
1000336-60-8	Ethyl 9-tetradecenoate	1.015
006114-18-7	(E)-9-Octadecenoic acid ethyl ester	1.008
010236-10-9	Ethyl 5-methylhexanoate	0.9937
003391-86-4	1-Octen-3-ol	0.9835
000105-37-3	Ethyl propionate	0.9792
000491-02-1	Cyclohexanol, 5-methyl-2-(1-methylethyl)-, (1.alpha.,2.alpha.,5.alpha.)-	0.9596
054546-22-4	Ethyl 9-hexadecenoate	0.9461
010348-47-7	ethyl 2-hydroxy-4-methylvalerate	0.9448
000112-12-9	2-Undecanone	0.9403
1000107-72-6	ETHYL (S)-(-)-LACTATE	0.9382
002050-01-3	Propanoic acid, 2-methyl-, 3-methylbutyl ester	0.9283
000108-95-2	Phenol	0.9038
000098-01-1	Furfural	0.9024
000493-01-6	Naphthalene, decahydro-, cis-	0.8966
002050-09-1	Pentanoic acid, 3-methylbutyl ester	0.8889
000111-70-6	Heptyl alcohol	0.8702
000624-13-5	Propyl octanoate	0.8674
068515-48-0	Phthalic acid, isobutyl nonyl ester	0.8646
000334-48-5	n-Decanoic acid	0.8575
091213-30-8	3-Nonenoic acid, ethyl ester	0.8521
000106-27-4	Butanoic acid, 3-methylbutyl ester	0.8514
000110-38-3	Ethyl caprate	0.8378

000096-76-4	Phenol, 2,4-bis(1,1-dimethylethyl)-	0.8308
000064-19-7	Acetic acid	0.8225
000091-16-7	Benzene, 1,2-dimethoxy-	0.8120
002785-89-9	4-Ethyl-2-methoxyphenol	0.8079
019132-06-0	(2S,3S)-(+)-2,3-Butanediol	0.7944
000513-86-0	2-Butanone, 3-hydroxy-	0.7937
000108-38-3	m-Xylene	0.7936
063366-65-4	9-Borabicyclo[3.3.1]nonane, 9-hydroxy-	0.7880
000109-52-4	Pentanoic acid	0.7847
000108-24-7	Acetic anhydride	0.7707
040348-72-9	Pentanoic acid, 2-hydroxy-4-methyl-, methyl ester	0.7699
000544-35-4	Linoleic acid ethyl ester	0.7627
023708-56-7	6-UNDECANOL	0.7432
000110-43-0	2-Heptanone	0.7430
000137-32-6	2-Methyl-1-butanol	0.7353
028024-16-0	ethyl isopentyl succinate	0.7324
039252-02-3	Furfuryl hexanoate	0.7143
006290-37-5	Hexanoic acid, 2-phenylethyl ester	0.6978
000123-92-2	Isoamyl acetate	0.6948
007619-08-1	9,12-Octadecadienoic acid ethyl ester	0.6935
000071-36-3	1-Butanol	0.6922
000093-51-6	2-Methoxy-4-methylphenol	0.6884
000110-19-0	Isobutyl acetate;	0.6699
000141-78-6	Ethyl acetate	0.6611
000628-99-9	2-Nonanol	0.6576
000078-92-2	2-Butanol	0.6560
001490-04-6	Cyclohexanol, 5-methyl-2-(1-methylethyl)-	0.6504
000100-52-7	Benzaldehyde	0.6480
000111-87-5	1-Octanol	0.6333
004457-71-0	1,5-Pentanediol, 3-methyl-	0.6307

000080-55-7	Ethyl-2-hydroxy-2-methyl propionat	0.6277
000071-41-0	1-Pentanol	0.6184
000099-96-7	Benzoic acid, 4-hydroxy-	0.6174
000096-17-3	2-Methyl butyraldehyde	0.6167
000585-24-0	ISOBUTYL LACTATE	0.6164
000078-70-6	Linalool	0.6151
000103-45-7	Phenethyl acetate	0.6115
000659-70-1	Butanoic acid, 3-methyl-, 3-methylbutyl ester	0.6041
000590-86-3	Isovaleraldehyd	0.6030
000097-62-1	ethyl 2-methylpropanoate	0.5889
000624-17-9	Diethyl azelate	0.5766
001468-39-9	Isovaleric anhydride	0.5766
001123-56-4	3,4-Dimethylbenzaldehyde	0.5703
000105-46-4	DL-sec-Butyl acetate	0.5662
054253-56-4	Formaldehyde, dipropylhydrazone	0.5647
000090-05-1	Phenol, 2-methoxy-	0.5629
000821-55-6	2-Nonanone	0.5591
000108-88-3	Toluene	0.5567
000104-61-0	¦Ã-Nonanolactone	0.5558
000540-18-1	Butanoic acid, pentyl ester	0.5545
018829-56-6	2-Nonenal, (E)-	0.5545
004887-30-3	Hexanoic acid, octyl ester	0.5545
1000406-42-6	Pentyl dotriacontyl ether	0.5545
000119-84-6	Hydrocoumarin	0.5545
001604-34-8	2-Undecanone, 6,10-dimethyl-	0.5545
002497-23-6	(E)-2-Decenyl acetate	0.5545
020296-29-1	3-Octanol	0.5544
000488-15-3	2-hydroxy-3-methylvaleric acid	0.5544
006512-99-8	Ethyl Oleate	0.5398
000071-23-8	1-Propanol	0.5350

005132-75-2	Heptanoic acid, octyl ester	0.5296
000091-17-8	Naphthalene, decahydro-	0.5247
002177-81-3	METHYL 2-METHYLHEXANOATE	0.5238
001195-09-1	2-Methoxy-5-methylphenol	0.5229
000123-86-4	Butyl acetate	0.5180
000079-20-9	Methylacetat	0.5151
000706-14-9	gamma-Decalactone	0.5071
000623-17-6	Furan-2-ylmethyl acetate	0.5050
002561-21-9	Acetic acid, trifluoro-, octyl ester	0.5033
000624-83-9	Isocyanatomethane	0.4988
002450-27-3	4-(Benzyloxy)-3-methoxy-2-nitrobenzaldehyde	0.4977
000628-68-2	Diethyleneglycol diacetate	0.4937
000627-90-7	Undecanoic acid, ethyl ester	0.4905
003221-61-2	Octane, 2-methyl-	0.4905
001472-09-9	Cyclopropane, octyl-	0.4905
000503-86-6	4H-Imidazol-4-one, 2-amino-1,5-dihydro-	0.4905
003618-12-0	Cyclodecene	0.4905
004747-07-3	Hexane, 1-methoxy-	0.4872
000104-76-7	2-Ethyl-1-hexanol	0.4859
000106-31-0	Butyric anhydride	0.4855
000592-84-7	Formic acid, butyl ester	0.4852
000110-45-2	Isopentyl formate	0.4827
006976-72-3	n-Heptyl hexanoate	0.4823
000713-95-1	.delta.-Dodecalactone	0.4823
042558-37-2	endo-2-Methylbicyclo[3.3.1]nonane	0.4823
000327-70-8	1-Pentanol, trifluoroacetate	0.4816
000687-47-8	Propanoic acid, 2-hydroxy-, ethyl ester, (L)-	0.4795
001630-94-0	Cyclopropane, 1,1-dimethyl-	0.4791
002050-23-9	Diethyl suberate	0.4746
000927-45-7	7-TRIDECANOL	0.4745

015764-16-6	2,4-Dimethyl benzaldehyd	0.4682
004412-91-3	3-Furanmethanol	0.4601
000625-44-5	Propane, 1-methoxy-2-methyl-	0.4596
053778-61-3	Oxetane, 2,3,4-trimethyl-	0.4596
020607-76-5	Butanal, propylhydrazone	0.4565
063958-52-1	(Z)-Oxacyclopentadec-6-en-2-one	0.4565
1000194-22-9	(3-Methyl-oxiran-2-yl)-methanol	0.4478
000589-40-2	Formic acid, 1-methylpropyl ester	0.4478
000332-77-4	Furan, 2,5-dihydro-2,5-dimethoxy-	0.4478
003282-53-9	Cyclohexene, 1-butyl-	0.4478
006092-54-2	Hexyl chloroformate	0.4469
067446-07-5	cis-5-Decen-1-yl acetate	0.4469
1000364-13-8	trans-4-Methylcyclohexanol, trifluoroacetate	0.4469
000111-13-7	2-Octanone	0.4432
000109-60-4	n-Propyl acetate	0.4386
059819-62-4	2-Propen-1-one, 1-cyclopropyl-	0.4379
1000193-07-8	3,7-Dimethyl-5-(phenylthio)octa-1,6-dien-3-ol	0.4379
147254-32-8	cis-4-Hydroxy-3-methyldecanoic acid lactone	0.4379
064275-73-6	5-Octen-1-ol, (Z)-	0.4379
004606-15-9	Benzeneacetic acid, propyl ester	0.4337
1000352-81-3	2,6-Dihydroxyacetophenone, 2TMS derivative	0.4334
000110-93-0	6-Methyl-5-hepten-2-one	0.4274
000818-49-5	4-Methyl-1-hexanol	0.4266
000123-39-7	N-Methyl formamid	0.4227
000629-41-4	1,8-Octanediol	0.4200
000123-20-6	Vinyl butyrate	0.4149
067801-33-6	3-(hydroxymethyl)nonan-2-one	0.4146
1000227-83-0	cis-2,5-Dimethylpiperazine	0.4111
1000282-83-1	Hexanoic acid, cyclobutyl ester	0.4111
000112-42-5	1-Undecanol	0.4078

000100-60-7	Cyclohexanamine, N-methyl-	0.4074
002051-49-2	Hexanoic acid, anhydride	0.4074
004411-89-6	2-Phenyl-2-butenal	0.4060
000645-08-9	3-Hydroxy-4-methoxybenzoic acid	0.4056
005978-70-1	(R)-(-)-2-Octanol	0.4018
000112-63-0	MEthyl linoleate	0.4004
010340-23-5	cis-3-Nonen-1-ol	0.4003
002235-83-8	5-Phenyl-2-pentanone	0.3938
000624-41-9	2-Methylbutyl acetate	0.3934
000122-70-3	Propanoic acid, 2-phenylethyl ester	0.3891
005779-94-2	2,5-Dimethyl benzaldehyde	0.3792
018433-98-2	2,5-Dimethyl-3-n-pentylpyrazine	0.3758
000108-82-7	2,6-Dimethyl-4-heptanol	0.3729
001569-60-4	6-Methyl-5-hepten-2-ol	0.3685
000105-43-1	3-Methylvaleric Acid	0.3680
000628-63-7	Amyl Acetate	0.3672
000077-68-9	3-hydroxy-2,2,4-trimethylpentyl isobutyrate	0.3663
014861-06-4	Vinyl but-2-enoate	0.3644
000106-90-1	2,3-Epoxypropyl acrylate	0.3629
000109-90-0	Ethyl Isocyanate	0.3557
1000315-39-3	decyl 2-phenylacetate	0.3551
052019-78-0	(S)-(+)-2-Hexanol	0.3511
000542-10-9	Ethylidene diacetate	0.3494
013748-90-8	Pentanoic acid, 2-hydroxy-4-methyl-, (.+/-.)-	0.3480
001070-34-4	Monoethyl succinate	0.3400
006033-23-4	(S)-(+)-2-Heptanol	0.3393
001467-79-4	Dimethylcyanamide	0.3334
?000111-87-5	1-Octanol	0.3143
031450-14-3	Ethyl?linolenate	0.3128
000527-53-7	1,2,3,5-TETRAMETHYL BENZENE	0.3110

000109-21-7	n-Butyl butanoate	0.3100
000628-28-4	1-Methoxybutane	0.3090
000110-63-4	1,4-Butanediol	0.3082
000532-55-8	Benzoyl isothiocyanat	0.3038
1000513-86-0	Acetoin	0.2951
007326-46-7	Tetrahydro-2-methyl-2-furanol	0.2938
000645-65-8	Imidazolyl-4-acetic acid	0.2913
123513-85-9	2,3-Butanediol	0.2839
000112-05-0	Nonanoic acid	0.2838
000627-98-5	5-Methyl-1-hexanol	0.2834
001655-03-4	1-(1-Cyclohexenyl)-1-propanone	0.2820
000111-21-7	ETHYLENEBIS-(2-OXYETHYL ACETATE)	0.2816
000106-68-3	3-Octanone	0.2789
002050-95-5	diisopentyl carbonate	0.2777
000624-54-4	pentyl propanoate?	0.2758
033467-76-4	Trans-2-Hepten-1-ol	0.2691
000141-32-2	Butyl acrylate	0.2669
000499-75-2	5-Isopropyl-2-methylphenol	0.2521
007452-79-1	Ethyl 2-methylbutanoate	0.2518
001066-42-8	dimethyl silanediol	0.2463
000124-13-0	Octanal	0.2249
000084-69-5	Diisobutylphthalat	0.2236
005137-52-0	Acetic acid, pentyl ester	0.2082
000141-06-0	Pentanoic acid, propyl ester	0.1634
006946-90-3	2-Hydroxyethyl hexanoate	0.1455

Table S3. The numbers of microbes at phyla, class, family, genus and ASV level in three aroma-type fermented grains.

**Taxonomic level**	**QX (n = 101)**		**NX (n = 98)**		**JX (n = 204)**	
	**Bacteria**	**Fungi**	**Bacteria**	**Fungi**	**Bacteria**	**Fungi**
Phylum	15	4	33	7	33	7
Class	23	14	73	25	79	25
Order	48	33	138	49	146	57
Family	80	46	209	103	231	115
Genus	170	86	416	151	528	187
ASV	811	648	1851	832	3032	1108

Table S4. Mantel test between microbial community and volatile chemical markers.

**Mental test**	**QX**		**NX**		**JX**	
	**Bacteria**	**Fungi**	**Bacteria**	**Fungi**	**Bacteria**	**Fungi**
P value	< 0.001	< 0.001	< 0.001	< 0.001	< 0.001	< 0.001

Table S5. Combinations of five-strain fermentation systems

**Combinations**	PK	SC	SP	ZB	TD
Combination 1	1	1	1	1	1
Combination 2	1	2	2	2	2
Combination 3	1	3	3	3	3
Combination 4	1	4	4	4	4
Combination 5	2	1	2	3	4

Combination 6	2	2	1	4	3
Combination 7	2	3	4	1	2
Combination 8	2	4	3	2	1
Combination 9	3	1	3	4	2
Combination 10	3	2	4	3	1
Combination 11	3	3	1	2	4
Combination 12	3	4	2	1	3
Combination 13	4	1	4	2	3
Combination 14	4	2	3	1	4
Combination 15	4	3	2	4	1
Combination 16	4	4	1	3	2

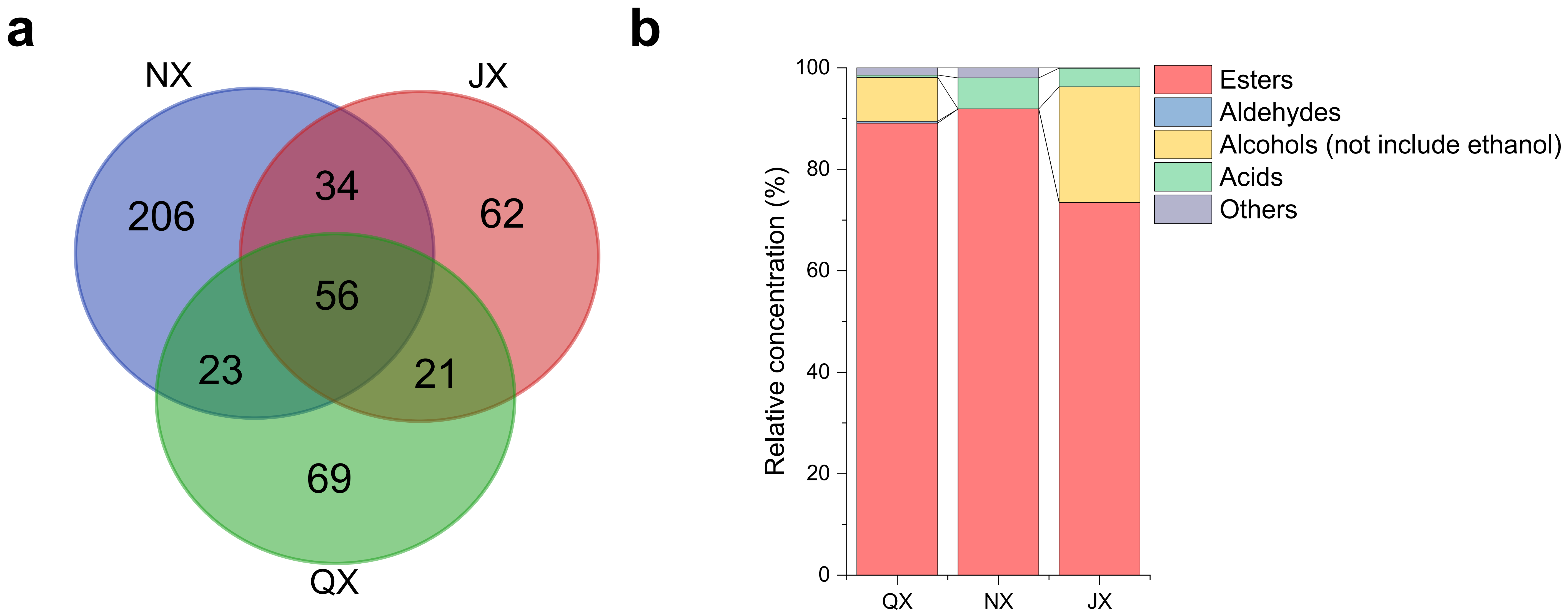
a
NX
JX
QX
206
34
62
56
23
21
69
b
Relative concentration (%)
0
20
40
60
80
100
QX
NX
JX
Esters
Aldehydes
Alcohols (not include ethanol)
Acids
Others

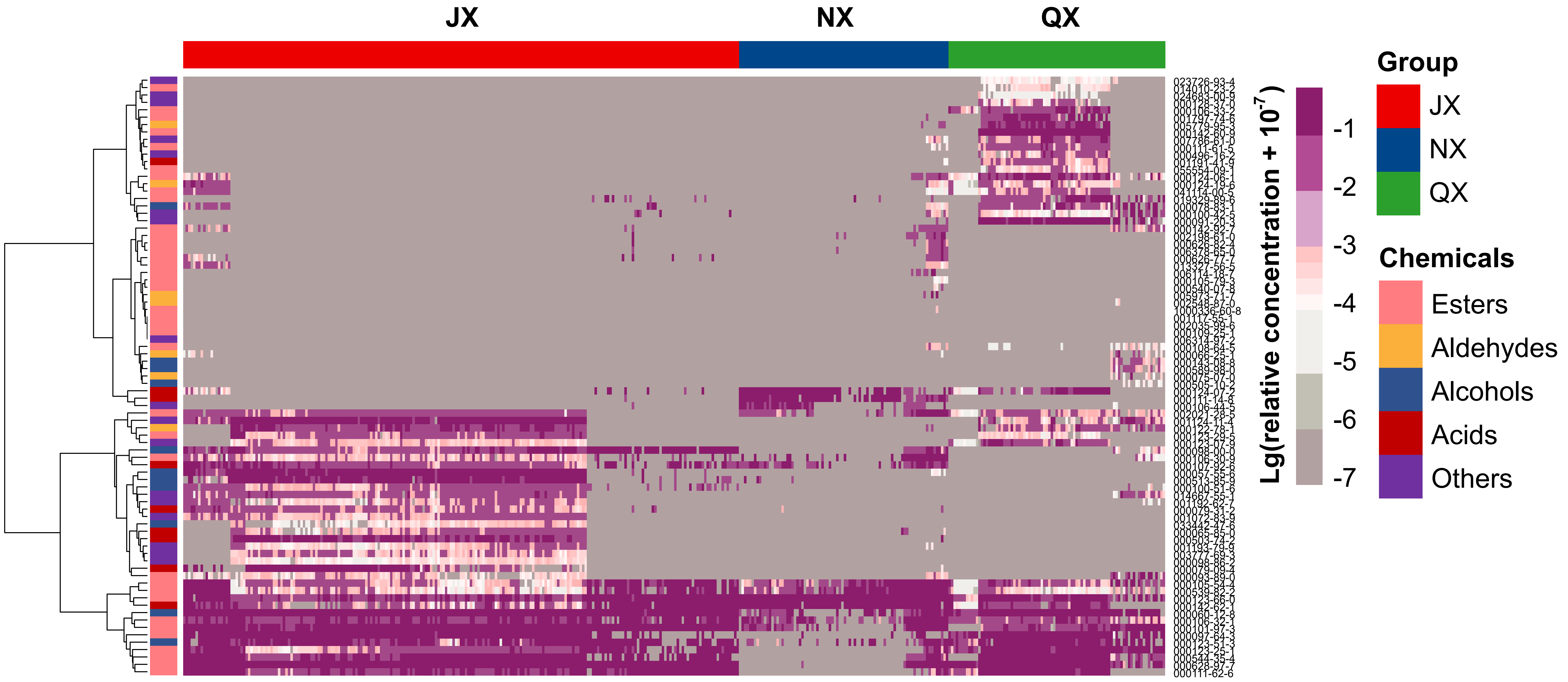
JX
NX
QX
Lg(relative concentration + 10^{-7})
-1
-2
-3
-4
-5
-6
-7
Group
JX
NX
QX
Chemicals
Esters
Aldehydes
Alcohols
Acids
Others

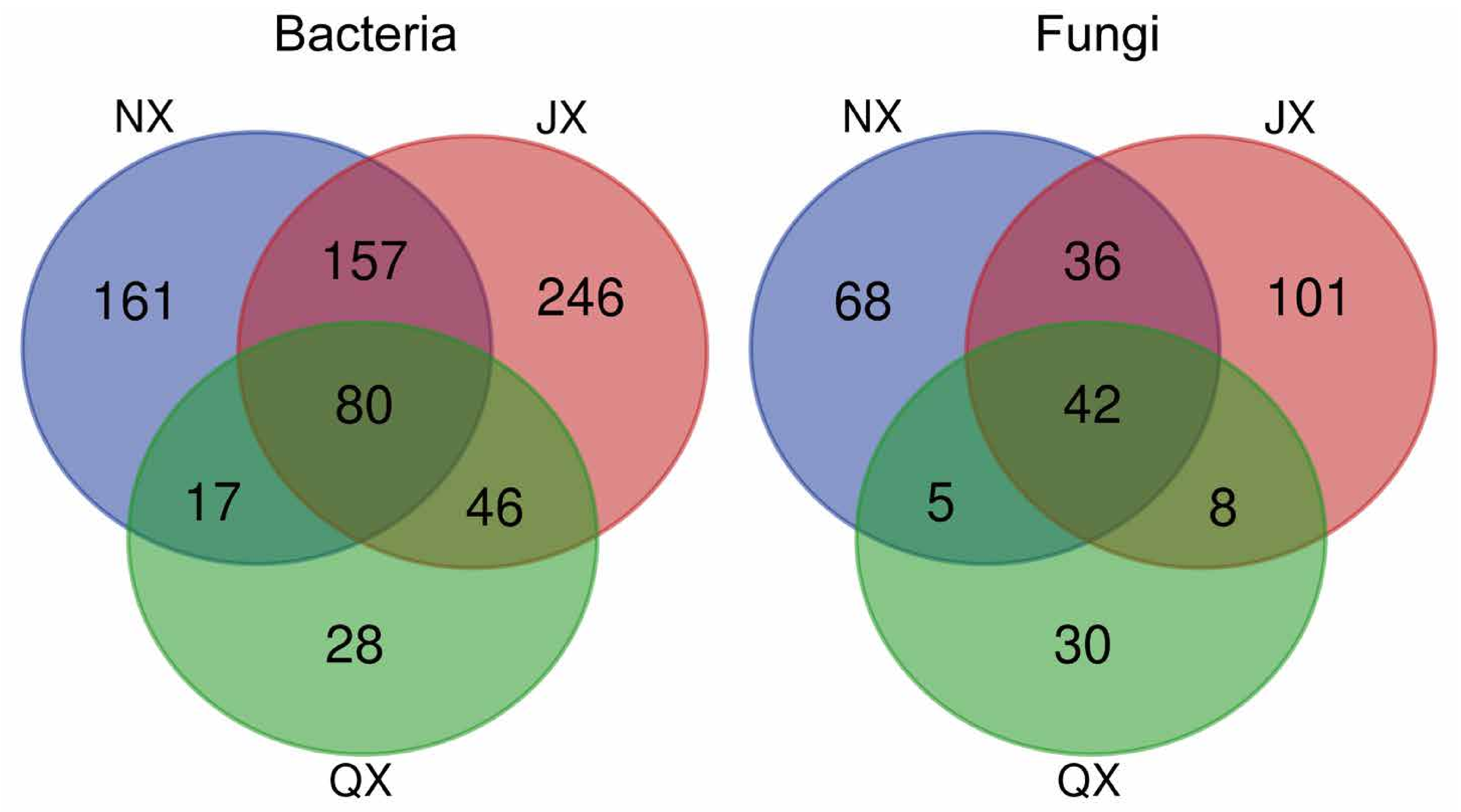
Bacteria
NX
JX
161
157
246
80
17
46
28
QX
Fungi
NX
JX
68
36
101
42
5
8
30
QX

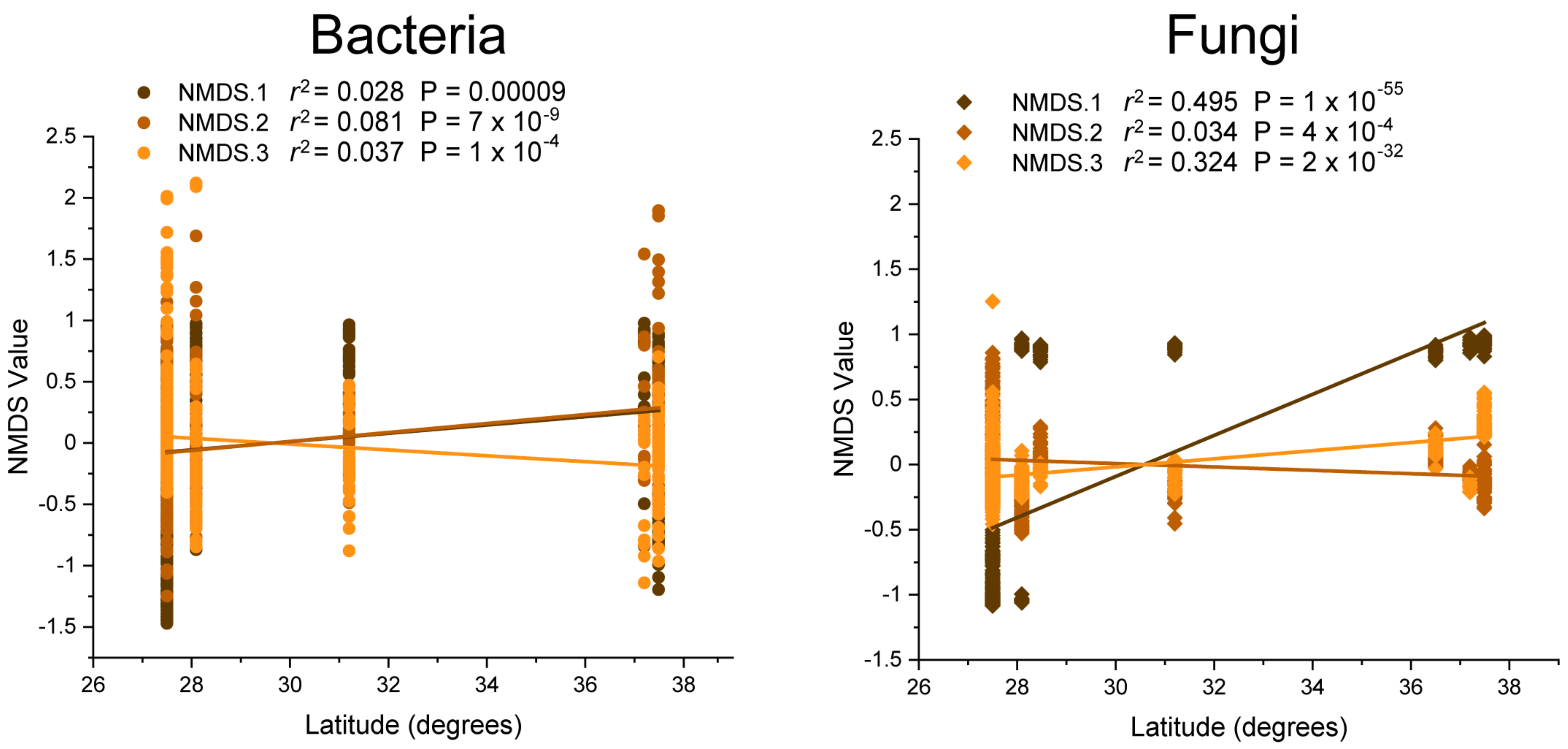

Bacteria
NMDS.1 r2 = 0.028 P = 0.00009
NMDS.2 r2 = 0.081 P = 7 x 10-9
NMDS.3 r2 = 0.037 P = 1 x 10-4
NMDS Value
Latitude (degrees)
Fungi
NMDS.1 r2 = 0.495 P = 1 x 10-55
NMDS.2 r2 = 0.034 P = 4 x 10-4
NMDS.3 r2 = 0.324 P = 2 x 10-32
NMDS Value
Latitude (degrees)

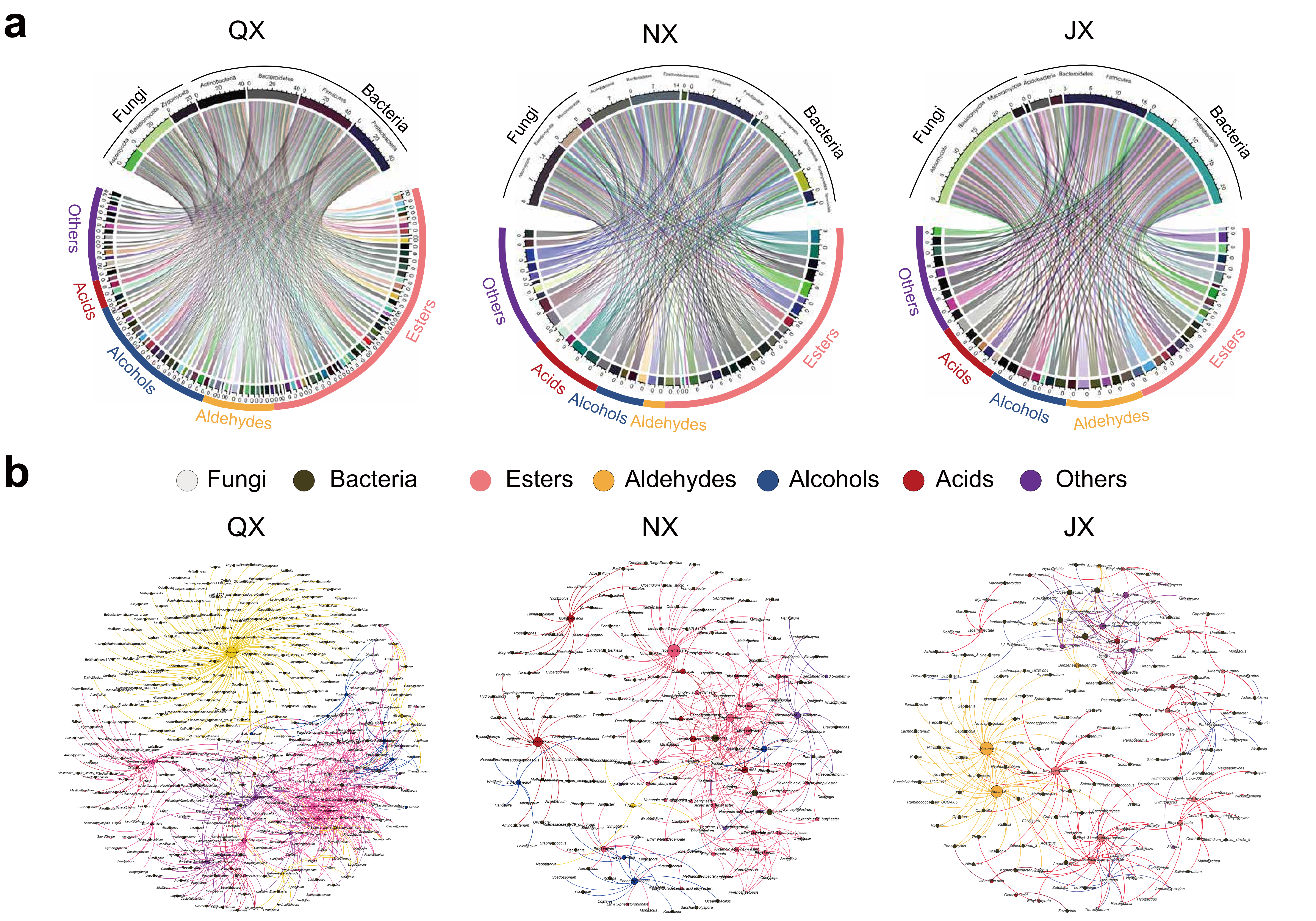
a
QX
NX
JX
Fungi
Bacteria
Others
Acids
Alcohols
Aldehydes
Esters
b
Fungi
Bacteria
Esters
Aldehydes
Alcohols
Acids
Others
QX
NX
JX

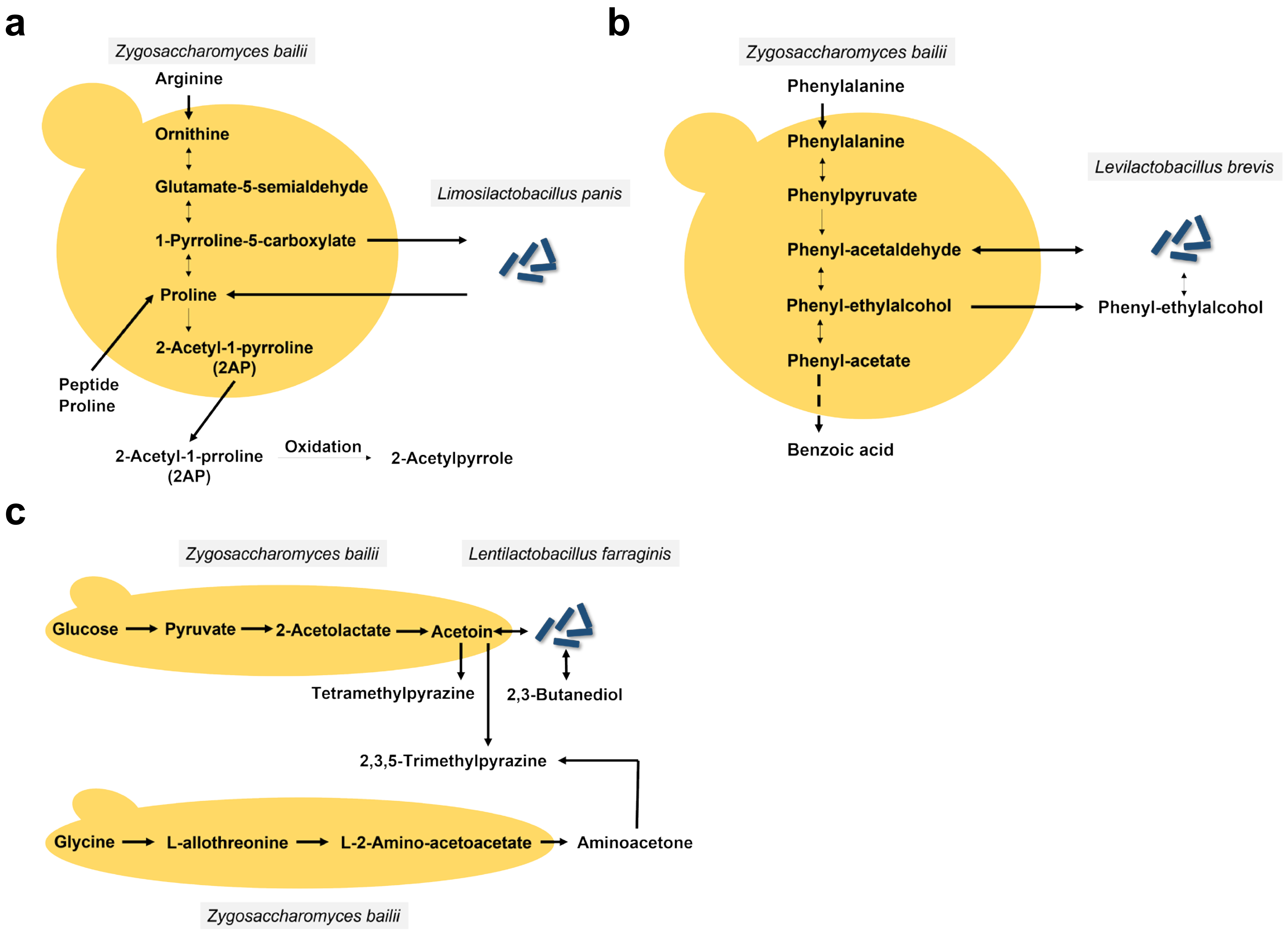
a
Zygosaccharomyces bailii
Arginine
Ornithine
Glutamate-5-semialdehyde
1-Pyrroline-5-carboxylate
Proline
2-Acetyl-1-pyrroline
(2AP)
Peptide
Proline
Limosilactobacillus panis
2-Acetyl-1-prroline
(2AP)
Oxidation
2-Acetylpyrrole
b
Zygosaccharomyces bailii
Phenylalanine
Phenylalanine
Phenylpyruvate
Phenyl-acetaldehyde
Phenyl-ethylalcohol
Phenyl-acetate
Benzoic acid
Levilactobacillus brevis
Phenyl-ethylalcohol
c
Zygosaccharomyces bailii
Lentilactobacillus farraginis
Glucose
Pyruvate
2-Acetolactate
Acetoin
Tetramethylpyrazine
2,3-Butanediol
2,3,5-Trimethylpyrazine
Glycine
L-allothreonine
L-2-Amino-acetoacetate
Aminoacetone
Zygosaccharomyces bailii

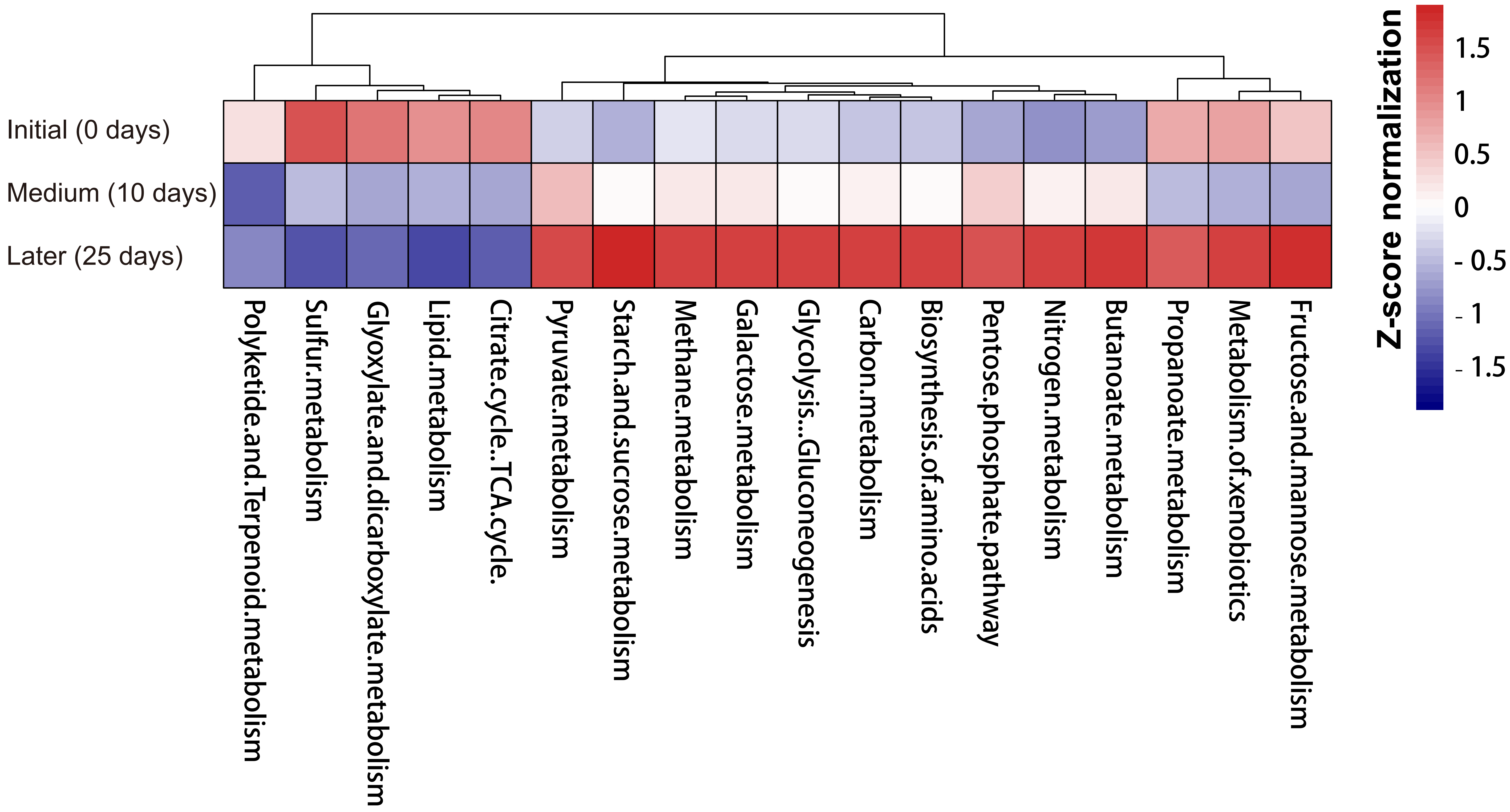

Initial (0 days)
Medium (10 days)
Later (25 days)
Polyketide.and.Terpenoid.metabolism
Sulfur.metabolism
Glyoxylate.and.dicarboxylate.metabolism
Lipid.metabolism
Citrate.cycle..TCA.cycle.
Pyruvate.metabolism
Starch.and.sucrose.metabolism
Methane.metabolism
Galactose.metabolism
Glycolysis...Gluconeogenesis
Carbon.metabolism
Biosynthesis.of.amino.acids
Pentose.phosphate.pathway
Nitrogen.metabolism
Butanoate.metabolism
Propanoate.metabolism
Metabolism.of.xenobiotics
Fructose.and.mannose.metabolism
Z-score normalization
1.5
1
0.5
0
- 0.5
- 1
- 1.5

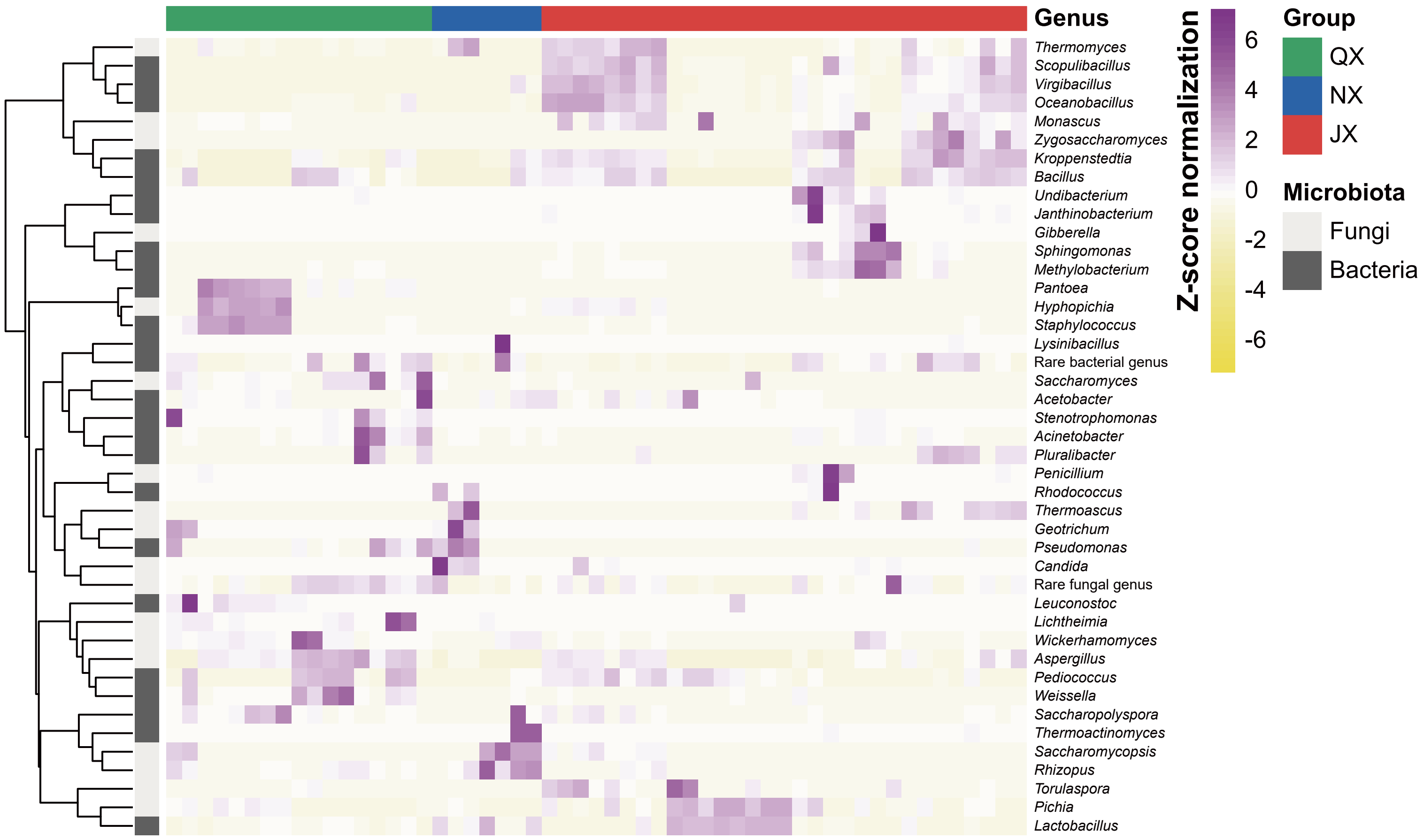
Genus
Thermomyces
Scopulibacillus
Virgibacillus
Oceanobacillus
Monascus
Zygosaccharomyces
Kroppenstedtia
Bacillus
Undibacterium
Janthinobacterium
Gibberella
Sphingomonas
Methylobacterium
Pantoea
Hyphopichia
Staphylococcus
Lysinibacillus
Rare bacterial genus
Saccharomyces
Acetobacter
Stenotrophomonas
Acinetobacter
Pluralibacter
Penicillium
Rhodococcus
Thermoascus
Geotrichum
Pseudomonas
Candida
Rare fungal genus
Leuconostoc
Lichtheimia
Wickerhamomyces
Aspergillus
Pediococcus
Weissella
Saccharopolyspora
Thermoactinomyces
Saccharomycopsis
Rhizopus
Torulaspora
Pichia
Lactobacillus
Z-score normalization
6
4
2
0
-2
-4
-6
Group
QX
NX
JX
Microbiota
Fungi
Bacteria

Saccharomyces cerevisiae + Fructilactobacillus fructivorans

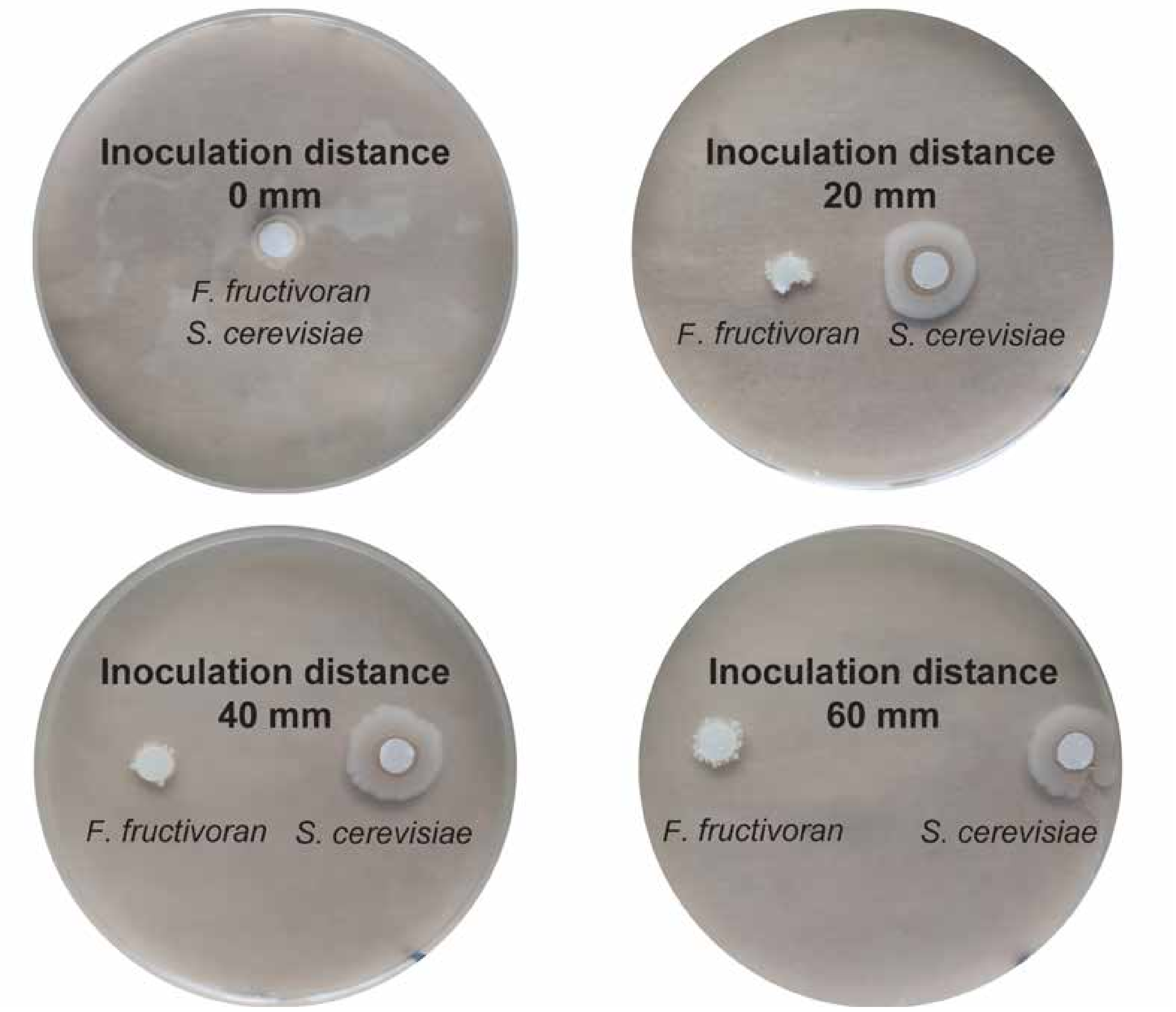

Supplement: Supplemental file 1 — Download SPECTRUM01844-22_Supp_S1_seq11.pdf, PDF file, 1.1 MB [file spectrum01844-22_supp_s1_seq11.pdf]
